# Polynucleotide Phosphorylase Regulates Multiple Virulence Factors and the Stabilities of Small RNAs RsmY/Z in *Pseudomonas aeruginosa*

**DOI:** 10.3389/fmicb.2016.00247

**Published:** 2016-03-02

**Authors:** Ronghao Chen, Yuding Weng, Feng Zhu, Yongxin Jin, Chang Liu, Xiaolei Pan, Bin Xia, Zhihui Cheng, Shouguang Jin, Weihui Wu

**Affiliations:** ^1^State Key Laboratory of Medicinal Chemical Biology, Key Laboratory of Molecular Microbiology and Technology of the Ministry of Education, Department of Microbiology, College of Life Sciences, Nankai UniversityTianjin, China; ^2^Department of Molecular Genetics and Microbiology, College of Medicine, University of FloridaGainesville, FL, USA

**Keywords:** PNPase, small RNA, gene regulation, pathogenesis, *P. aeruginosa*

## Abstract

Post-transcriptional regulation enables bacteria to quickly response to environmental stresses. Polynucleotide phosphorylase (PNPase), which contains an N-terminal catalytic core and C-terminal RNA binding KH-S1 domains, is involved in RNA processing. Here we demonstrate that in *Pseudomonas aeruginosa* the KH-S1 domains of PNPase are required for the type III secretion system (T3SS) and bacterial virulence. Transcriptome analysis revealed a pleiotropic role of PNPase in gene regulation. Particularly, the RNA level of *exsA* was decreased in the ΔKH-S1 mutant, which was responsible for the reduced T3SS expression. Meanwhile, the pilus biosynthesis genes were down regulated and the type VI secretion system (T6SS) genes were up regulated in the ΔKH-S1 mutant, which were caused by increased levels of small RNAs, RsmY, and RsmZ. Further studies revealed that deletion of the KH-S1 domains did not affect the transcription of RsmY/Z, but increased their stabilities. An *in vivo* pull-down and *in vitro* electrophoretic mobility shift assay (EMSA) demonstrated a direct interaction between RsmY/Z and the KH-S1 fragment. Overall, this study reveals the roles of PNPase in the regulation of virulence factors and stabilities of small RNAs in *P. aeruginosa*.

## Introduction

PNPase is a phosphate-dependent 3–5′ exonuclease with homologs identified in bacteria and eukaryotes (Guarneros and Portier, 1991; Symmons et al., 2000). PNPase consists of two RNase PH (pnp1 and pnp2) domains at the N-terminus and two RNA binding domains, KH and S1 on the C-terminus (Bermúdez-Cruz et al., 2005; Briani et al., 2007; Fernández-Ramírez et al., 2010). In bacteria, a small proportion of PNPase forms a large multi-protein complex with ribonuclease E (RNase E), a RNA helicase RhlB and a glycolytic enzyme enolase, named the RNA degradosome, which plays an important role in RNA processing (Miczak et al., 1996; Vanzo et al., 1998; Khemici et al., 2004; AJ, 2007; Nurmohamed et al., 2009).

PNPase is involved in bacterial response to environmental stresses (Rosenzweig and Chopra, 2013). For example, PNPase is required for the growth at low temperatures of *E. coli, Yersinia pestis*, and *Yersinia pseudotuberculosis, Salmonella enterica, Campylobacter jejuni, Bacillus subtilis*, and *Staphylococcus aureus* (Jones et al., 1987; Wang and Bechhofer, 1996; Goverde et al., 1998; Clements et al., 2002; Rosenzweig et al., 2005, 2007; Anderson and Dunman, 2009; Haddad et al., 2009). In addition, PNPase is involved in the regulation of virulence factors in several pathogenic bacteria, including *Y. pestis, Y. pseudotuberculosis, S. enterica*, and *Dichelobacter nodosus* (Rosenzweig and Chopra, 2013). In *Yersinia*, PNPase is required for the T3SS (Rosenzweig and Schesser, 2007), which is a highly conserved protein delivery system in various Gram-negative animal and plant pathogens (Sheahan and Isberg, 2015). The T3SS injects effector proteins into host cell cytosol, interfering with host cell signaling and other cellular processes, which facilitates bacterial pathogenesis (Hueck, 1998). In *Yersinia*, the effect of PNPase on the T3SS requires its S1 domain but is independent of its ribonuclease activity (Rosenzweig et al., 2005). However, the molecular mechanism remains elusive. In *S. typhimurium*, the wild type strain causes acute systemic infection in an intraperitoneal challenge assay, whereas the *pnp* mutant establishes a persistent infection in mice, suggesting a role of PNPase in the regulation of different sets of virulence factors (Clements et al., 2002).

So far, PNPase has been found to control gene expression mainly through three mechanisms: degradation of mRNA, affecting translation, and modulating sRNA stability. For example, PNPase autoregulates its own expression through RNase III dependent and independent mechanisms in *E. coli* (Wong et al., 2013; Carzaniga et al., 2015). In the RNase III dependent pathway, the *pnp* mRNA is processed by RNase III, followed by degradation in a PNPase dependent mechanism (Robert-Le Meur and Portier, 1994; Jarrige et al., 2001). In the RNase III independent pathway, PNPase binds to the 5′ untranslated region (5′UTR) of its own mRNA through its KH-S1 domains, which excludes the binding of ribosomal protein S1 and inhibits the translation (Carzaniga et al., 2015). In the cold shock response, the role of PNPase is to degrade unnecessary cold shock protein transcripts and resume growth after cold shock in both *E. coli* and *Yersinia enterocolitica* (Neuhaus et al., 2000; Polissi et al., 2003). Other than mRNAs, PNPase is involved in the degradation of small RNAs (sRNAs) that do not associate with RNA chaperone Hfq in *E. coli* (Andrade et al., 2012). However, PNPase was also found to be required for the stability of several sRNAs including RyhB, SgrS, and CyaR in *E. coli* through an unknown mechanism (De Lay and Gottesman, 2011).

Previously, we found that a *pnp*::Tn mutant was defective in the secretion of a T3SS effector protein ExoS in *Pseudomonas aeruginosa* (Li et al., 2013). *P. aeruginosa* is a versatile Gram-negative bacterium, which causes acute and chronic infections in humans (Stover et al., 2000; Driscoll et al., 2007). Virulence factors, including T3SS and motility play important roles in acute infections (Sadikot et al., 2005). During chronic infections, *P. aeruginosa* forms biofilm, in which bacteria grow inside an extracellular matrix mainly composed of polysaccharide, DNA and protein (Deretic et al., 1995; Sadikot et al., 2005). High level expression of type VI secretion system (T6SS) HSI-I is often associated with biofilm formation during chronic infection (Aubert et al., 2008; Khajanchi et al., 2009). It has been demonstrated that the T6SS plays a major role in killing target bacterial cells through translocation of toxic effector proteins in a cell–cell contact-dependent process (MacIntyre et al., 2010; Russell et al., 2011).

In *P. aeruginosa*, two small RNAs, RsmY, and RsmZ (RsmY/Z), reciprocally regulate acute and chronic infection associated virulence factors. RsmY/Z directly bind to RsmA and suppress its function (Brencic et al., 2009; Bordi et al., 2010). RsmA is a global post-transcriptional regulatory protein, which binds to a GGA motif in untranslated region of target mRNAs and represses their translation (Mulcahy et al., 2008). The direct target of RsmA includes mRNAs of T6SS genes *fha1* and *tssA1*, as well as *pslA*, a gene involved in biofilm matrix exopolysaccharide synthesis (Brencic and Lory, 2009; Irie et al., 2010). Meanwhile, RsmA positively regulates the T3SS and the type VI pili through an unknown mechanism (Brencic and Lory, 2009).

The levels of RsmY/Z are controlled at the transcriptional and posttranscriptional level. The two-component regulatory system GacS-GacA directly controls the transcription of RsmY/Z (Brencic et al., 2009). Interaction between RsmY and Hfq protects RsmY from degradation by RNase E (Sonnleitner et al., 2006; Sorger-Domenigg et al., 2007).

In this study, we demonstrate that PNPase is essential for the T3SS and pathogenesis in *P. aeruginosa*. Transcriptome analysis reveals that PNPase controls multiple virulence factors, including T6SS and pilus biosynthesis genes. And we demonstrate a direct interaction between PNPase and RsmY/Z whereby PNPase controls the stability of these sRNAs and subsequently the expression of T6SS and pilus biosynthesis genes. These results provide a new insight into the regulatory mechanism of PNPase in *P. aeruginosa*.

## Materials and methods

### Bacterial strains, plasmids, and growth conditions

The strains and plasmids used in this study are listed in Table S1. All strains were cultured in LB broth or L-agar (LA) at 37°C. Antibiotics were used at the following concentrations: for *E. coli*, ampicillin at 100 μg/ml, kanamycin at 50 μg/ml, gentamicin at 15 μg/ml; for *P. aeruginosa*, carbenicillin at 150 μg/ml, gentamicin at 50 μg/ml, tetracycline at 50 μg/ml.

### Murine acute pneumonia model

All animal experiments complied with Nankai University and Chinese national guidelines regarding the use of animals in research. The protocol was approved by the institutional animal care and use committee of the college of life sciences of Nankai University with permit number: NK-04-2012. Overnight bacterial culture was diluted in fresh LB and grown to an optical density at 600 nm (OD_600_) of 1.0. The bacterial cells were collected and resuspended in phosphate-buffered saline (PBS). Female BALB/c mice (6–8 weeks old) were anesthetized with 0.1 ml of 7.5% chloral hydrate injected intraperitoneally. Then the mice were inoculated intranasally with 2 × 10^7^ CFU bacteria. Twelve hours post infection, the mice were sacrificed. The lungs were isolated and homogenized in 1% proteose peptone. The bacterial loads were determined by serial dilutions and plating. Statistical analysis was performed with the GraphPad Prism software.

### Cytotoxicity assay

Bacterial cytotoxicity was determined by measuring detachment of mammalian cells after bacterial infection as described previously (Li et al., 2013). 1.2 × 10^5^ HeLa cells were seeded into each well of a 24-well plate and cultured in Dulbecco's modified Eagle medium (DMEM) with 10% (vol/vol) heat-inactivated fetal bovine serum (hiFBS), penicillin G (100U/ml), and streptomycin (100 μg/ml) at 37°C with 5% CO_2_ for 18 h. The medium was replaced with antibiotic free DMEM with 10% hiFBS 1 h before bacterial infection. Overnight bacterial culture was diluted 50-fold in fresh LB and grown to an OD_600_ of 1.0. Bacteria were washed twice and resuspended in PBS. HeLa cells were infected with indicated strains at a multiplicity of infection (MOI) of 20. After 3 h, the medium in each well was removed. Cells remaining attached were washed twice with PBS and stained with 0.1% crystal violet for 15 min. Then, each well was washed twice with water. For quantification, the stained crystal violet was dissolved in 95% ethanol and measured at the wavelength of 590 nm.

### Western blotting

Over night bacterial culture was diluted 1:100 in LB or 1:30 in LB supplemented with 5 mM EGTA, and cultured for 3.5 h. The supernatant and pellet were separated by centrifugation. Samples from equal numbers of bacteria were loaded onto a 12% sodium dodecyl sulfate-polyacrylamide gel (SDS-PAGE). The proteins were transferred onto a polyvinylidene difluoride (PVDF) membrane and probed with a rabbit polyclonal antibody against ExoS or a mouse monoclonal antibody against FLAG (Sigma). Signals were detected with the ECL-plus kit (Millipore).

### RNA extraction and real time PCR

Bacteria were grown in LB medium to indicated growth phases. Total RNA was isolated with an RNA prep Pure cell/Bacteria Kit (Tiangen Biotec). cDNA was synthesized with a PrimeScript Reverse Transcriptase and random primers (Takara). The cDNA was mixed with indicated primers and FastStart Essential DNA Green Master (Roche). The pyrroline-5-carboxylate reductase coding gene *proC* was used as an internal control.

### Transcriptome sequencing and analysis

The Transcriptome sequencing and analysis were performed by GENEWIZ (Suzhou, China). Briefly, total RNA of each sample was quantified and qualified by an Agilent 2100 Bioanalyzer (Agilent Technologies). One microgram total RNA with RIN value above seven was used for library preparation. Large ribosomal RNA was depleted from bacterial total RNA using RiboMinus Bacteria Module (Invitrogen) and the rRNA-depleted mRNA was then fragmented, and primed with random primers. Pair-end index libraries were constructed according to the manufacturer's protocol (NEBNext. Ultra. Directional RNA Library Prep Kit for Illumina). The RNA expression analysis was based on the annotations of PAO1 (www.pseudomonas.com). The RSEM software (V 1.2.15) was used to align the input reads against the reference gene with Bowtie2 and expression values were calculated using the FPKM (fragments per kilobase of transcript per million reads) method. The software edger (V3.4.2) (Bioconductor) was used to calculate *p*-values.

### RNA stability analysis

Bacteria were grown to an OD_600_ of 1.0 and treated with 100 μg/ml rifampicin. At each indicated time point, same volume of bacteria was taken and the bacterial concentration was determined by serial dilution and plating. Next, each sample was mixed with equal numbers of *gfp* expressing *E. coli* cells. Total RNA was purified and the levels of RsmY/Z were analyzed with real time PCR. The *gfp* RNA level in each sample was used as an internal control for normalization.

### Twitching motility

The twitching motility was assayed on 1% LB agar. Each strain was inoculated in the agar by stabbing with a sharp toothpick. The plates were incubated at 37°C for 18 h. The twitching zones were visualized by staining with 0.1% crystal violet.

### Purification of protein and detection of associated RNA

The C-terminus His-tagged full length PNPase or KH-S1 fragment was constructed in pMMB67EH and transferred into wild type PAK constitutively expressing a *gfp* gene. Bacteria containing either one of the plasmids were grown to an OD_600_ of 0.8 and expression of the His-tagged protein was induced by the addition of 1 mM IPTG for 3 h. Bacteria from 100 mL culture were collected by centrifugation and resuspended in 1.5 ml lysis buffer (500 mM NaCl, 50 mM Tris, 20 mM imidazole, pH 7.9) with recombinant RNase inhibitor (RRI, Takara) and lysed by sonication. After centrifugation, the supernatant was incubated with 30 μl Ni-NTA agarose beads (Qiagen) at 4°C for 1 h. Then the beads were washed 5 times with 1 ml lysis buffer, and incubated with 30 μl elution buffer (500 mM NaCl, 50 mM Tris, 250 mM imidazole, pH 7.9) for 10 min. After centrifugation, protein and RNAs in the supernatant were collected and subjected to RNA purification with an RNA prep Pure cell/Bacteria Kit (Tiangen Biotec). The purified RNA was analyzed by real time PCR. The *gfp* RNA was used as the reference to determine the relative level of each sRNA.

### *In vitro* transcription and RNA gel mobility shift assay

The sRNA transcripts were synthesized from PCR products using the Riboprobe System-T7 (Promega) according to the manufacturer's instructions. The primers used in PCR are shown in supporting information (Table S2). The RNAs were purified with an RNA prep Pure cell/ Bacteria Kit (Tiangen Biotec) and refolded by heating at 90°C for 10 min followed by natural cooling at room temperature for 30 min. One nano mole sRNA was incubated with indicated amount of purified KH-S1-His protein in 20 μl binding buffer [10 mM Tris-HCl, pH 7.5; 50 mM KCl, 5 mM MgCl_2_, 10% glycerol, 1 U recombinant RNase inhibitor (Takara)] for 30 min at room temperature. Fifteen microliters of each sample was loaded onto a non-denaturing 7% polyacrylamide gel. Electrophoresis was performed at 100 V for 150 min with 0.5 × TBE buffer (Tris-borate-ethylenediaminetetraacetic acid) at 4°C. The RNA bands were observed by staining with ethidium bromide (Takara) for 15 min.

## Results

### PNPase is required for ExoS expression and cytotoxicity

**Figure 1:**
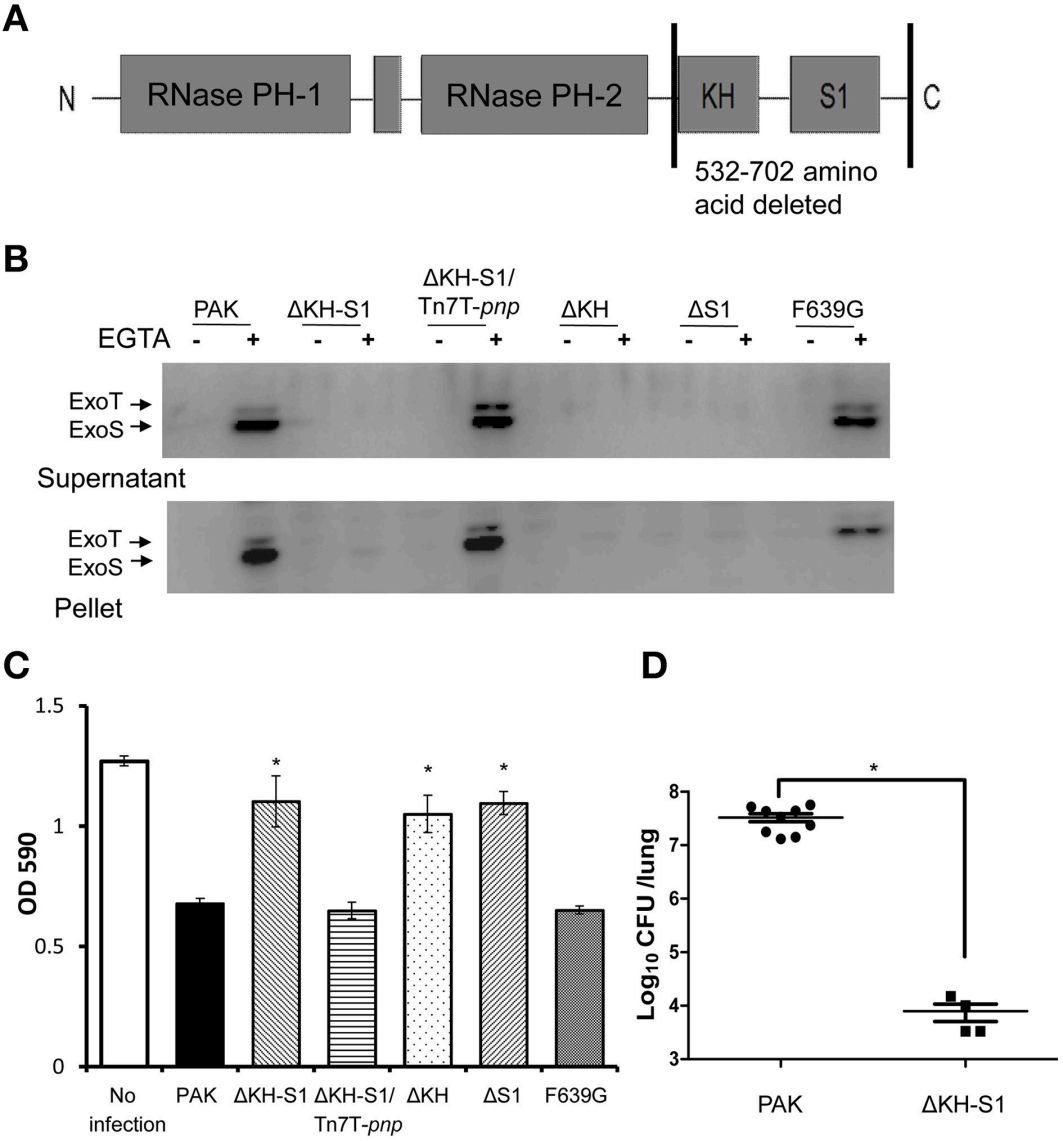
**Role of PNPase in the expression of T3SS and pathogenesis. (A)** Structure of the PNPase protein in *P. aeruginosa*. **(B)** Secretion and expression of ExoS in indicated strains. Bacteria were grown to an OD_600_ of 1.0 in LB with or without 5 mM EGTA. Lysates from equivalent bacterial cells were loaded onto SDS-PAGE gels and probed with the ExoS antibody. The ExoS antibody cross-recognizes another T3SS effector ExoT due to a high sequence homology between the two proteins. **(C)** Cytotoxicity of the indicated strains. HeLa cells were infected with the indicated strains at a MOI of 20. After 3 h, cells attached to the 24-well plate were quantified by crystal violet staining. Each assay was done in triplicates, and the error bars indicate standard deviations. ^*^p < 0.05 compared to PAK by Student's *t*-test. **(D)** Bacterial load in the lungs of mice infected with *P. aeruginosa*. 6–8 weeks old female BALB/c mice were inoculated with 2 × 10^7^ CFU wild-type PAK or the ΔKH-S1 mutant. Twelve hours post infection, the mice were sacrificed, and the lungs were isolated and homogenized. The bacteria loads were determined by serial dilution and plating. Long lines represent medians and shot lines represent standard errors of the means (SEM). ^*^p < 0.05, by Mann Whitney test.

In the *P. aeruginosa* genome, the PNPase coding gene *pnp* is 2106 base pairs (bp) long. Our previous study demonstrated that transposon (Tn) insertion at the 1655 bp of the *pnp* gene in a wild type strain PAK resulted in reduced ExoS secretion (Li et al., 2013). In order to investigate the role of PNPase in T3SS, we firstly tried to delete the entire *pnp* gene in wild type PAK. However, we were unable to obtain the mutant after multiple attempts. Then, we constructed a mutant with the deletion of nucleotides 1655–2106, which includes the coding region of the KH and S1 domains of PNPase (Figure 1A), and designated ΔKH-S1. As shown in Figure 1B, the expression of ExoS was lower in this mutant under T3SS inducing condition (in the presence of EGTA). For complementation, the full length *pnp* gene driven by its native promoter was inserted into the chromosome by a mini-Tn7 vector (Choi and Schweizer, 2006), which restored the expression and secretion of ExoS (Figure 1B).

In *P. aeruginosa*, the T3SS plays a major role in killing host cells. To examine the role of PNPase in cytotoxicity, HeLa cells were infected with PAK, the ΔKH-S1 mutant and the complemented strain at a MOI of 20. When infected with PAK, majority of the cells were rounded and detached 3 h post infection. Deletion of the KH and S1 domains reduced the cytotoxicity, which was restored by the complementation with a full length *pnp* gene (Figure 1C). These results suggest that loss of the KH-S1 domains of PNPase results in defective T3SS in *P. aeruginosa*.

### PNPase is essential for the bacterial virulence in a mouse acute pneumonia model

The T3SS is activated during infection and plays an important role in the pathogenesis in acute infections (Roy-Burman et al., 2001; Hauser, 2009; Howell et al., 2013). To evaluate the role of PNPase in virulence, BALB/c mice were infected intranasally with 2 × 10^7^ CFU of PAK or the ΔKH-S1 mutant. 12 h post infection, the mice were sacrificed, and the bacterial load in each lung was enumerated. Compared to the wild type PAK, significantly less ΔKH-S1 mutant bacteria were recovered from the lungs (Figure 1D), suggesting an essential role of PNPase in the colonization by *P. aeruginosa*.

### Role of the KH and S1 domains in the regulation of T3SS

In *E. coli*, PNPase negatively regulates its own mRNA stability and translation (Wong et al., 2013; Carzaniga et al., 2015). Therefore, deletion of the KH-S1 domains in *P. aeruginosa* might result in an up regulation of the remaining N-terminal catalytic core domains in the chromosome and lead to defective T3SS. To test the expression level of the N-terminal domains, we cloned the coding region with its native promoter and fused it with a His tag on its C-terminus, and designated it as NTD-His (Figure S1A). The construct was inserted into the chromosomes of PAK and the ΔKH-S1 mutant. As shown in Figure S1B, the expression levels of the NTD-His were similar between the wild type PAK and the ΔKH-S1 mutant, suggesting that the defective T3SS is not due to an increase of the N-terminal catalytic core domains.

In *Yersinia*, the S1 domain but not the PNPase ribonuclease activity is required for the T3SS (Rosenzweig et al., 2005). Of the S1 domain, the F639 residue is conserved and plays an integral role in RNA binding. Replacement of the F639 residue with glycine reduces the PNPase catalytic activity and T3SS expression in *Yersinia* (Jarrige et al., 2002; Schubert et al., 2004; Rosenzweig et al., 2007). To test the role of these two domains, we complemented the ΔKH-S1 mutant with a *pnp* gene without either the KH or the S1 domain, or with a F639G mutation. As shown in Figures 1B,C, PNPase without the KH or S1 domain was unable to restore the T3SS and cytotoxicity, suggesting an essential role of both the KH and S1 domains. However, we cannot exclude the possibility that deletion of the KH domain affects the correct folding of the S1 domain. The F639G mutation had no influence on the T3SS expression and cytotoxicity (Figures 1B,C). In addition, complementation with a fragment containing the KH-S1 domains did not restore the T3SS expression in theΔKH-S1 mutant (Figure S2). These results indicate a difference in the PNPases in *Yersinia* and *P. aeruginosa*.

### Identification of genes affected by PNPase

To further understand the role of PNPase in T3SS and possibly other genes' expression, we performed transcriptome analyses (RNA-seq) on wild type PAK and the ΔKH-S1 mutant. The two strains were grown to log phase and total RNAs were purified. Compared to wild type PAK, expression of 309 genes were altered, with 182 genes down regulated, and 127 genes up regulated in the ΔKH-S1 mutant (Table S3). Consistent with aforementioned results, mRNA levels of the T3SS genes were reduced in the ΔKH-S1 mutant (Table 1). Besides, the type IV pilus biosynthesis genes were down regulated (Table 1) while the T6SS and biofilm matrix exopolysaccharide genes were up regulated. Additionally, the RNA levels of ribosome proteins were increased in the absence of PNPase (Table S3). The full data set has been deposited in the NCBI SRA, with the Study accession number SRP069795.). These results revealed that the PNPase has a global impact on mRNA levels in *P. aeruginosa*.

**Table 1:** **Genes of altered expression in the ΔKH-S1 mutant compared to wild-type PAK**.

**Gene category and designation**	**Gene**	**Gene function**	**Fold change (ΔKH-S1/WT)**	***P*-value**
**TYPE III SECRETION GENES**				
PA0044	*exoT*	exoenzyme T	0.159907	0.000010
PA1691	*pscT*	translocation protein in type III secretion	0.532172	0.049187
PA1693	*pscR*	type III secretion system protein	0.207216	0.000117
PA1696	*pscO*	translocation protein in type III secretion	0.216924	0.000260
PA1698	*popN*	type III secretion outer membrane protein PopN	0.294441	0.001205
PA1703	*pcrD*	type III secretion apparatus protein PcrD	0.352609	0.003461
PA1704	*pcrR*	transcriptional regulator PcrR	0.320746	0.006457
PA1705	*pcrG*	regulator in type III secretion	0.404124	0.010673
PA1706	*pcrV*	type III secretion protein PcrV	0.316899	0.001794
PA1708	*popB*	translocator protein PopB	0.216440	0.000110
PA1709	*popD*	translocator outer membrane protein PopD	0.188098	0.000038
PA1710	*exsC*	exoenzyme S synthesis protein C	0.480077	0.022071
PA1711	*exsE*	ExsE protein	0.466334	0.021247
PA1713	*exsA*	transcriptional regulator ExsA	0.387678	0.006181
PA1714	*exsD*	ExsD protein	0.344493	0.003011
PA1715	*pscB*	type III export apparatus protein	0.241351	0.000354
PA1716	*pscC*	type III secretion outer membrane protein PscC	0.313879	0.001609
PA1717	*pscD*	type III export protein PscD	0.27541	0.000721
PA1718	*pscE*	type III export protein PscE	0.07945	5.95E-06
PA1719	*pscF*	type III export protein PscF	0.11164	7.59E-07
PA1720	*pscG*	type III export protein PscG	0.11603	1.24E-06
PA1721	*pscH*	type III export protein PscH	0.15169	1.26E-05
PA1722	*pscI*	type III export protein PscI	0.19506	6.60E-05
PA1723	*pscJ*	type III export protein PscJ	0.24424	0.000288
PA1724	*pscK*	type III export protein PscK	0.21932	0.000216
PA1725	*pscL*	type III secretion system protein	0.28892	0.001065
PA2191	*exoY*	adenylate cyclase	0.293252	0.001068
PA3841	*exoS*	exoenzyme S	0.141582	0.000004
**TYPE VI SECRETION GENES**				
PA0086	*tagJ1*	Protein secretion/export apparatus	4.467933	0.019493
PA0087	*tssE1*	protein secretion by the type VI secretion system	3.514331	0.047076
PA0090	*clpV1*	ClpV1 protein	3.501512	0.049148
PA0091	*vgrG1*	VgrG protein	4.604618	0.012635
**BIOFILM**				
PA2240	*pslJ*	protein PslJ	3.902872	0.030241
PA3059	*pelF*	protein PelF	5.52508	0.004936
PA3060	*pelE*	protein PelE	4.03186	0.034716
PA3063	*pelB*	protein PelB	5.902027	0.002977
PA3064	*pelA*	PelA protein	4.589041	0.013927
**PILI BIOGENESIS GENES**				
PA0411	*pilJ*	twitching motility protein PilJ	0.433964	0.011866
PA0412	*pilK*	methyltransferase PilK	0.319080	0.001878
PA0396	*pilU*	twitching motility protein PilU	0.3314	0.002268
PA4525	*pilA*	type IV fimbrial PilA	0.51187	0.030077
PA4527	*pilC*	type IV fimbrial biogenesis protein PilC	0.54672	0.041833
PA4547	*pilR*	two-component response regulator PilR	0.46878	0.019024
PA4550	*fimU*	type IV fimbrial biogenesis protein FimU	0.39812	0.007478
PA4551	*pilV*	type IV fimbrial biogenesis protein PilV	0.35586	0.003935
PA4552	*pilW*	type IV fimbrial biogenesis protein PilW	0.36734	0.004521
PA4553	*pilX*	type IV fimbrial biogenesis protein PilX	0.43412	0.012812
PA4554	*pilY1*	type IV fimbrial biogenesis protein PilY1	0.40549	0.008012
PA4555	*pilY2*	type IV fimbrial biogenesis protein PilY2	0.37192	0.005566
PA4556	*pilE*	type IV fimbrial biogenesis protein PilE	0.35542	0.003868
PA5040	*pilQ*	type IV fimbrial biogenesis outer membrane protein	0.358040	0.003676
PA5041	*pilP*	type IV fimbrial biogenesis protein PilP	0.386421	0.006050
PA5042	*pilO*	type IV fimbrial biogenesis protein PilO	0.462024	0.017119
PA5043	*pilN*	type IV fimbrial biogenesis protein PilN	0.51759	0.031694
PA5044	*pilM*	type IV fimbrial biogenesis protein PilM	0.50816	0.028528

### Over expression of *exsA* restores the T3SS expression in the ΔKH-S1 mutant

**Figure 2:**
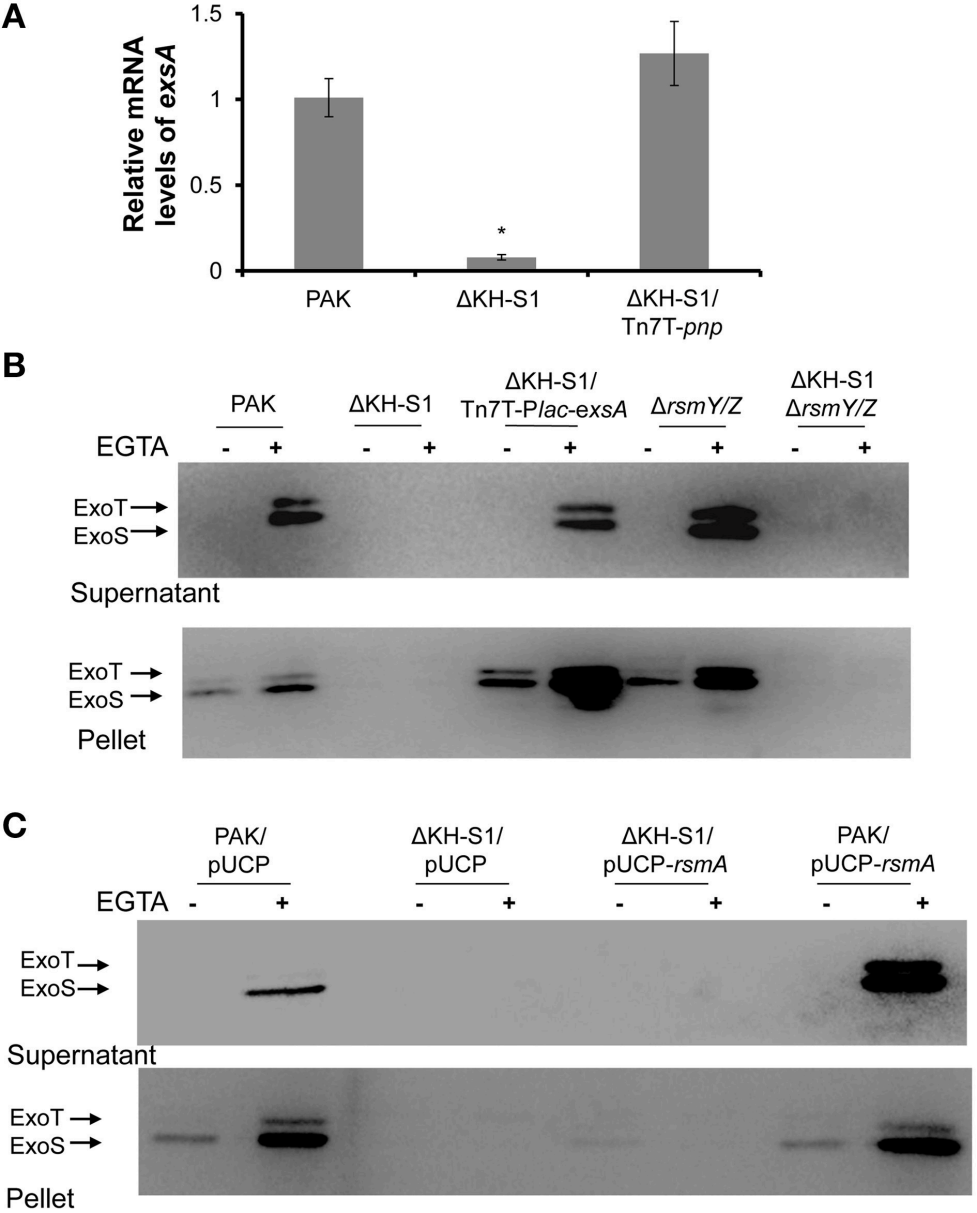
**RsmY/Z and RsmA are not involved in PNPase-mediated regulation of T3SS. (A)** Relative mRNA levels of *exsA* in indicated strains. Bacteria were grown to an OD_600_ of 1.0. Total RNA was isolated and the *exsA* mRNA levels were determined by real time PCR with *proC* as an internal control. Each assay was done in triplicates, and the error bars indicate standard deviations. ^*^p < 0.05 compared to PAK by Student's *t*-test. **(B,C)** Secretion and expression of ExoS in indicated strains. Bacteria were grown to an OD_600_ around 1.0 in LB with or without 5 mM EGTA. Intracellular and secreted ExoS were separated by centrifugation. Samples from equivalent bacterial cells were loaded into SDS-PAGE gels and probed with antibody against ExoS.

ExsA directly controls the expression of T3SS genes through binding to their promoters (Hauser, 2009). Our transcriptome analysis reveals a 61% decrease of the *exsA* mRNA in the ΔKH-S1 mutant (Table 1), which was confirmed by real time PCR analysis (Figure 2A). To test if the defective T3SS is due to reduced expression of ExsA, we inserted a *lac* promoter driven *exsA* gene into the chromosome by the mini-Tn7 vector (Choi and Schweizer, 2006; Sun et al., 2014). As shown in Figure 2B, over expression of ExsA restored the expression and secretion of ExoS in the ΔKH-S1 mutant. These results suggest that PNPase affects the T3SS through ExsA.

### Twitching motility is affected by the *PNP* mutation

**Figure 3:**
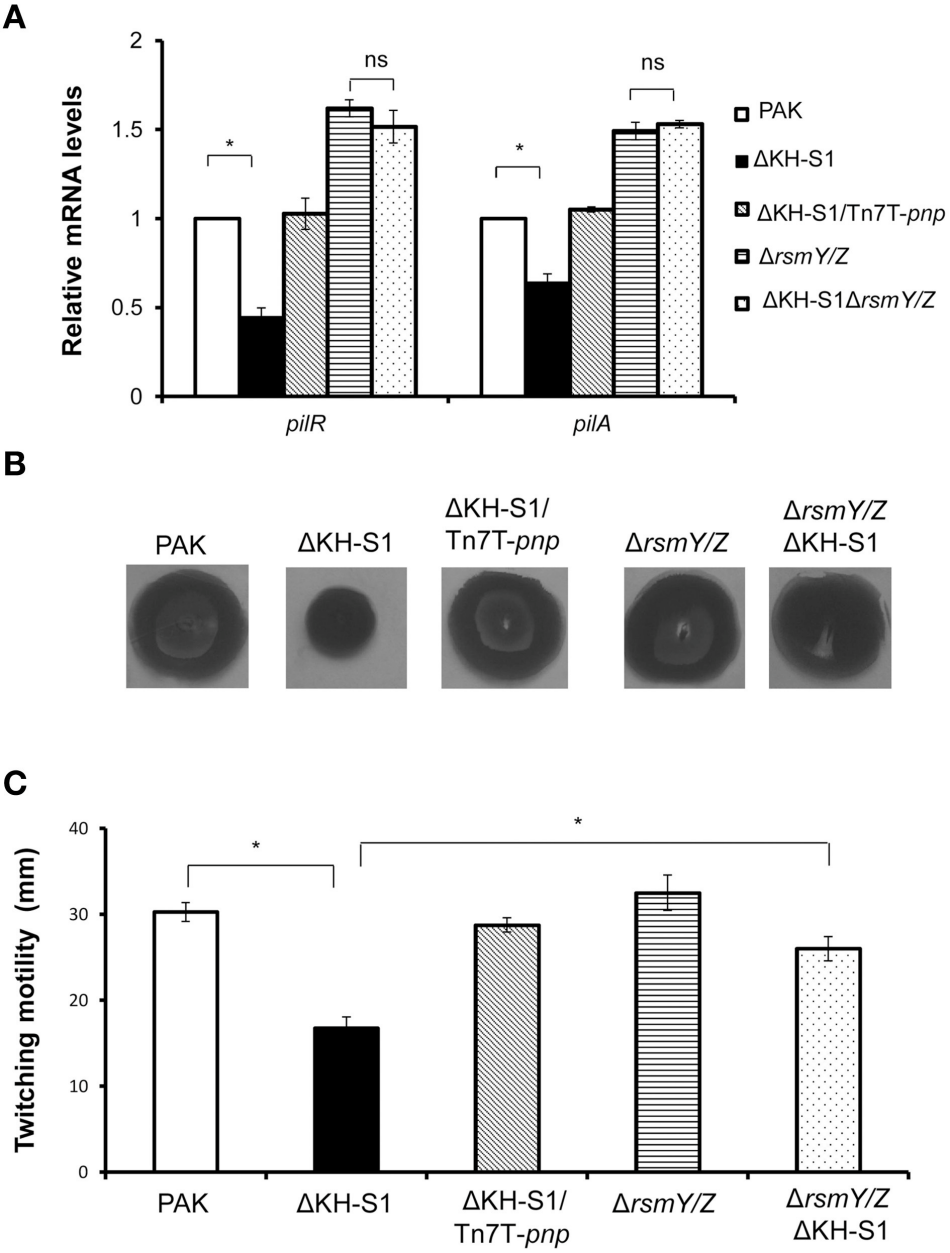
**PNPase regulates the type IV pili through RsmY/Z. (A)** Bacteria were grown to an OD_600_ of 1.0. Total RNAs were collected and the relative mRNA levels of *pilR* and *pilA* were determined by real time PCR. Each assay was done in triplicates, and the error bars indicate standard deviations. ^*^p < 0.05 compared to wild type PAK by Student's *t*-test. **(B)** Twitching motilities of the indicated strains were assayed on 1% LB agar. The twitching zones were visualized by staining with 0.1% crystal violet**. (C)** Diameters of the twitching zones of indicated strains. The values represent the average diameters from three independent experiments. The error bars indicate standard deviations. ^*^p < 0.05 compared to wild type PAK by Student's *t*-test.

As shown in Table 1, the mRNA levels of type IV pilus biosynthesis genes were decreased in the ΔKH-S1 mutant. Real time PCR results confirmed the down regulation of type IV pilus regulatory genes *pilR* and structural gene *pilA* in the ΔKH-S1 mutant. Complementation with a *pnp* gene restored the expression of these two genes (Figure 3A). Consistent with the gene expression pattern, the ΔKH-S1 mutant displayed defective twitching motility, which was restored in the complementation strain (Figures 3B,C). These results suggest a role of PNPase in the regulation of twitching motility.

### T6SS is up regulated in the ΔKH-S1 mutant

**Figure 4:**
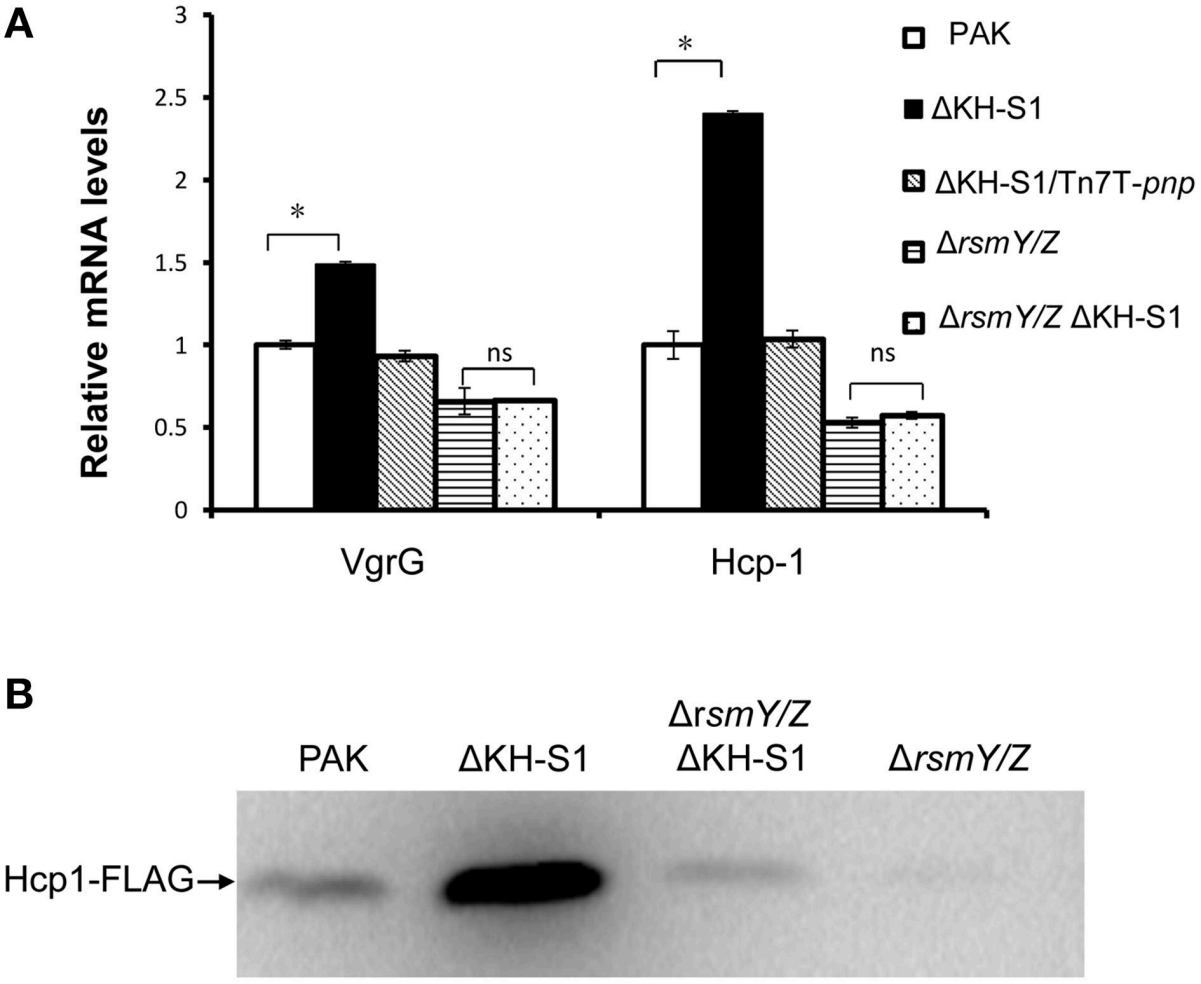
**PNPase regulates the T6SS expression through RsmY/Z. (A)** Relative mRNA levels of the T6SS genes in indicated strains. Each assay was done in triplicates, and the error bars indicate standard deviations. ^*^p < 0.05 compared to wild type PAK by Student's *t*-test; ns, not significant. **(B)** Levels of Hcp-1 protein in indicated strains. Strains with an *hcp1*-FLAG in their chromosomes were grown for 7 h in LB. Samples from equivalent numbers of bacterial cells were loaded onto a SDS-PAGE gel and probed with an anti-FLAG antibody.

In our transcriptome analysis, the mRNA levels of four T6SS HSI-I genes were significantly increased in the ΔKH-S1 mutant (Table 1). Real time PCR verified the up regulation of *vgrG* and *hcp-1* (Figure 4A). To further confirm the expression level, a *hcp1* gene driven by its native promoter was tagged with a FLAG and inserted into the chromosomes of indicated strains. Consistent with the mRNA level, the Hcp-1 protein level was increased in the ΔKH-S1 mutant (Figure 4B).

### Increased levels of RsmY and RsmZ are responsible for the altered expression of pili, T6SS, but not T3SS in the *PNP* mutant strain

**Figure 5:**
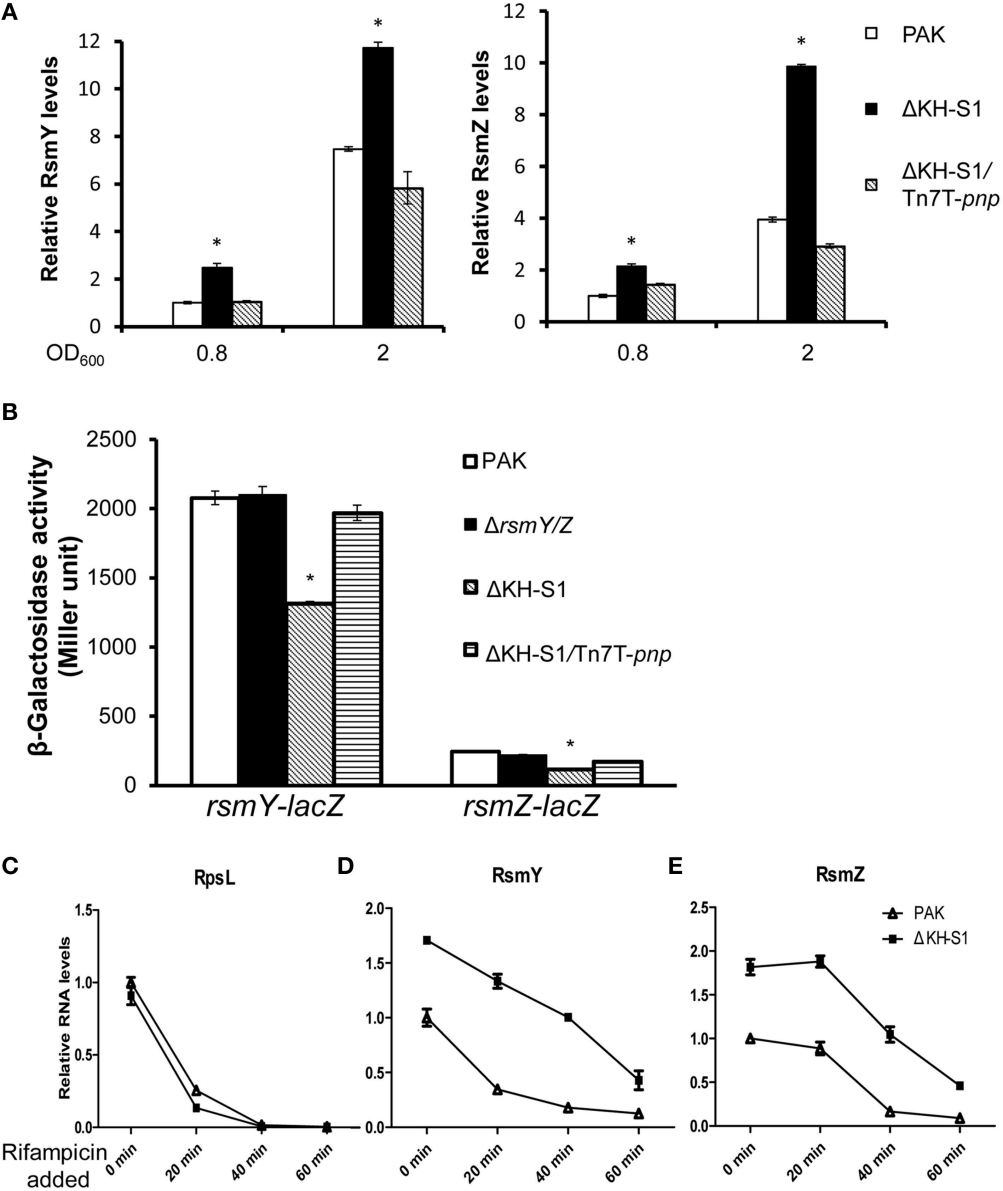
**PNPase controls the stabilities of RsmY/Z. (A)** Levels of the RsmY and RsmZ in indicated strains. Bacteria were grown to an OD_600_ = 0.8 or 2 in LB. Total RNAs were collected and the relative levels of RsmY and RsmZ were determined with real time PCR. The RNA levels of *proC* were used as internal controls. ^*^p < 0.05 compared to wild type PAK by Student's *t*-test. **(B)** Expression of RsmY and RsmZ in wild type PAK and the ΔKH-S1 mutant. Bacteria containing *rsmY*-*lacZ* or *rsmZ*-*lacZ* transcriptional fusion were grown to the similar OD_600_ of 2.0 and subjected to β-galactosidase assays. Each assay was done in triplicates, and the error bars indicate standard deviations. ^*^p < 0.05 compared to wild type PAK by Student's *t*-test. Degradation of RpsL **(C)**, RsmY **(D)**, and RsmZ **(E)** in wild type PAK and the ΔKH-S1 mutant. Bacterial cells with or without rifampicin treatment were spiked with equal numbers of *gfp* expressing *E. coli* cells. Total RNA was purified and the relative RNA levels were determined by real time PCR. The *gfp* RNA level in each sample was used as an internal control for normalization.

The down regulation of T3SS and pilus as well as up regulation of T6SS genes resemble the phenotypes of a *rsmA* mutant (Brencic and Lory, 2009). However, the RsmA mRNA levels were similar between wild type PAK and the ΔKH-S1 mutant (Figure S3). Since the function of RsmA is antagonized by small RNAs RsmY and RsmZ, we examined the levels of these sRNAs. Indeed, the RsmY/Z levels were higher in the ΔKH-S1 mutant at both late exponential and stationary phases, while complementation with a *pnp* gene restored the RNA levels (Figure 5A).

To test the role of RsmY/Z in the ΔKH-S1 mutant, we constructed a ΔKH-S1Δ*rsmY*Δ*rsm*Z triple mutant. Expression and secretion of ExoS were similar between the triple mutant and the ΔKH-S1 mutant (Figure 2B). And over expression of RsmA had no effect on the expression of ExoS in the ΔKH-S1 mutant (Figure 2C). These results suggest that RsmA and RsmY/Z are not involved in the altered expression of T3SS in the ΔKH-S1 mutant.

However, deletion of *rsmY/Z* restored the expression of pilus biosynthesis genes and twitching motility (Figures 3A–C), as well as the expression of *vgrG* and *hcp-1* in the ΔKH-S1 mutant (Figures 4A,B). Therefore, increased levels of RsmY/Z are responsible for the suppression of twitching motility and up regulation of T6SS genes.

### PNPase controls the stabilities of RsmY/Z

The higher levels of RsmY/Z in the ΔKH-S1 mutant might be due to increased transcription or stability. To test these possibilities, we firstly constructed *rsmY-lacZ* and *rsmZ-lacZ* transcriptional fusions. Compared to wild type PAK, the LacZ levels were slightly lower in the ΔKH-S1 mutant (Figure 5B). In addition, the mRNA levels of LadS, GacS and GacA, which are known to positively regulate RsmY/Z (Brencic et al., 2009), were slightly decreased in the *pnp* mutant (Figure S3). These results suggest that the higher levels of RsmY/Z are not due to increased transcription.

**Figure 6:**
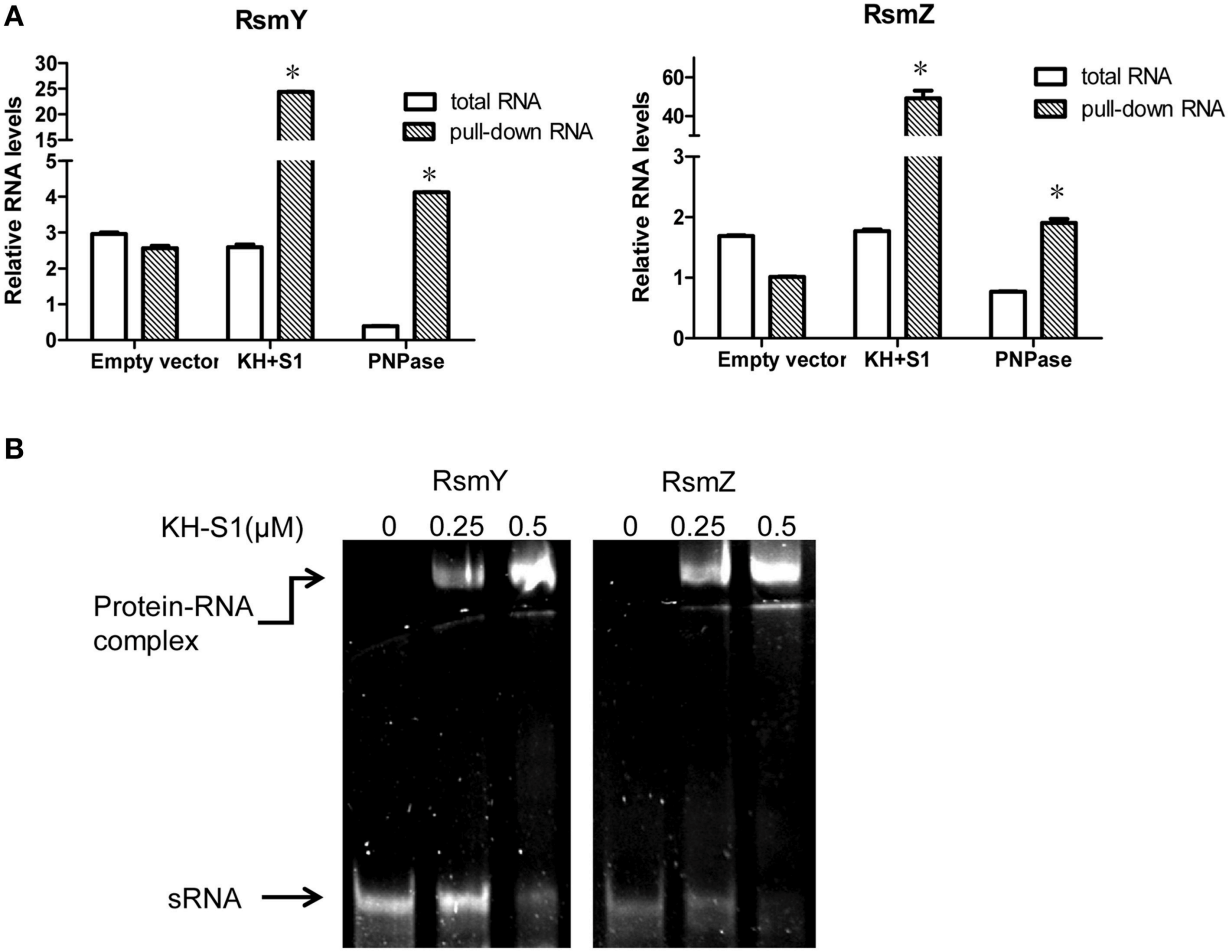
**PNPase directly binds to RsmY/Z through the KH-S1 domain. (A)** Levels of RsmY/Z in bacterial cells or co-purified with His-tagged PNPase or KH-S1 fragment. Expression of a His-tagged full length PNPase or KH-S1 fragment on plasmid pMMB67EH was induced by IPTG in wild type PAK carrying a *gfp* gene. The cellular levels of RsmY/Z were determined with real time PCR. The His-tagged proteins were purified by nickel affinity chromatography and associated RNAs were purified and analyzed with real time PCR. The *gfp* RNA level in each sample was used as an internal control. Each assay was done in triplicates, and the error bars indicate standard deviations. ^*^p < 0.05 compared to total RNA by Student's *t*-test. **(B)** Formation of RNA-protein complexes in the presence of RsmY/Z and KH-S1-6His fragments. The RsmY, RsmZ RNAs were generated by *in vitro* transcription. RNA gel mobility shift assay was performed in a non-denaturing polyacrylamide gel. Positions of free RNA and RNA-protein complexes are indicated by arrows.

Next, we compared the stabilities of RsmY/Z in wild type PAK and the ΔKH-S1 mutant. Bacteria were grown to an OD_600_ of 1.0, and 100 μg/ml rifampicin was added to block RNA transcription. After 20, 40, or 60 min, total RNA was extracted and examined with real time PCR. The ribosomal RNA (rRNA) RpsL levels were similar between PAK and the ΔKH-S1 mutant with or without rifampicin treatment, suggesting a similar degradation rate (Figure 5C). The RsmY level in the ΔKH-S1 mutant was 1.76–fold of that in PAK without rifampicin treatment, whereas 20 min after rifampicin treatment, the differences rose to 3.5-fold, and then dropped to around 3-folds after 40 or 60 min (Figure 5D). As for the RsmZ levels, the difference between the ΔKH-S1 mutant and PAK rose from 1.8-fold without rifampicin treatment to 2-, 4-, and 5-fold after 20, 40, and 60 min treatment, respectively (Figure 5E). These results suggest increased stability of the sRNAs in the ΔKH-S1 mutant. Furthermore, over expression of the PNPase in wild type PAK reduced the levels of RsmY/Z (Figures 6A,B), suggesting a role of PNPase in the degradation of RsmY/Z.

It has been reported that the RNA chaperone Hfq binds to and stabilizes RsmY (Sonnleitner et al., 2006; Sorger-Domenigg et al., 2007). To test whether the increased stability of RsmY is due to up regulation of Hfq, we examined the Hfq level by real time PCR. As shown in Figure S4, the mRNA levels of Hfq were the same between the wild type strain and the ΔKH-S1 mutant. Therefore, the increased stabilization of RsmY/Z in the ΔKH-S1 mutant is not due to higher level of Hfq.

### PNPase directly binds to RsmY/Z

The RNA binding domains carried by PNPase and its role in controlling RsmY/Z suggest a possible interaction between PNPase and RsmY/Z. To test this possibility, we carried out a PNPase-RNA or KH-S1-RNA co-purification experiment. Expression of the C-terminus His-tagged full length PNPase or KH-S1 fragment on plasmid pMMB67EH was induced by IPTG in wild type PAK carrying a *gfp* gene. The His-tagged proteins were purified by nickel affinity chromatography and the associated RNAs were further isolated and analyzed by real time PCR. The levels of *gfp* RNA were used as reference to calculate the relative levels of RsmY/Z. If the relative level of RsmY or RsmZ is higher in the nickel affinity chromatography purified sample than that purified from bacterial cells, we consider there is enrichment, which indicates interaction between the protein and sRNA. As shown in Figure 6A, RsmY/Z were enriched with the purified His-tagged PNPase or KH-S1 fragment, although induction of full length PNPase reduced the cellular levels of RsmY/Z. Meanwhile, no enrichment of sRNA was observed in the empty vector control, suggesting a specific interaction between the KH-S1 fragment and RsmY/Z.

To test whether the co-purification of RsmY/Z and the KH-S1 domain is due to direct interaction, we performed a RNA gel mobility shift assay. *In vitro* transcribed RsmY/Z were incubated with purified His tagged KH-S1 fragment. As shown in Figure 6B, the KH-S1 fragment was able to retard both sRNAs, suggesting a direct interaction between the KH-S1 fragment and RsmY/Z. In combination, our results suggest that PNPase regulates the stability of RsmY/Z via direct PNPase–RNA binding.

## Discussion

In this study, we studied the relationship between the KH-S1 domains of PNPase and the virulence factors as well as sRNAs in *P. aeruginosa*. In our experiments, we were unable to delete the full length *pnp* gene in wild type PAK even after multiple attempts. Ectopic expression of a *pnp* gene driven by a regulatable *lac* promoter enabled us to delete the *pnp* gene on the chromosome. In the presence of IPTG, the mutant grew as fast as the wild type strain. However, in the absence of IPTG, the strain grew more slowly (Figure S5). We suspect that the leaky expression of the *pnp* gene driven by the *lac* promoter might support the growth of the mutant. Therefore, it is likely that the RNase PH domains play an important role for the growth of *P. aeruginosa* under normal growth condition.

RNA metabolism and processing are essential for bacterial survival and response to environmental stresses. In *E. coli*, the expression of PNPase is up regulated upon cold shock (Jones et al., 1987; Cairrão et al., 2003). And PNPase is required for the growth at low temperature for a number of bacteria (Jones et al., 1987; Wang and Bechhofer, 1996; Goverde et al., 1998; Clements et al., 2002; Rosenzweig et al., 2007; Anderson and Dunman, 2009). We found that the *P. aeruginosa* ΔKH-S1 mutant was unable to grow at 16°C in LB medium (data not shown), suggesting a similar role of PNPase in *P. aeruginosa*.

Besides cold shock, PNPase is involved in the regulation of virulence factors in several pathogenic bacteria (Rosenzweig and Chopra, 2013). In *Y. pseudotuberculosis* and *Y. pestis*, it was shown that the Δ*pnp* strain was less virulent (Rosenzweig et al., 2005, 2007). In *S. enterica*, PNPase promotes acute infection (Clements et al., 2002). Here in this study, we found that the KH-S1 domains of PNPase are required for the expression of acute infection associated virulence factors in *P. aeruginosa*, including T3SS and pili. Similarly, PNPase is required for the T3SS in *Y. pseudotuberculosis* and the S1 RNA binding domain of PNPase restored the T3SS activity (Rosenzweig et al., 2005, 2007). However, PNPase is required for the secretion rather than expression of the T3SS in *Yersinia* (Rosenzweig et al., 2005, 2007). In fact, the transcription of T3SS genes was at relatively higher level in the Δ*pnp* strain (Rosenzweig et al., 2007). Similar to *Yersinia*, the expression T3SS genes were relatively higher in Δ*pnp* mutant compared to the wild-type strain in *S. enterica* (Rosenzweig et al., 2007; Rosenzweig and Chopra, 2013). It seems that the roles of PNPase in the regulation of T3SS are different in *Yersinia, Salmonella* and *P. aeruginosa*, which might be due to different regulatory pathways or mechanisms.

Our transcriptomic analysis on the *P. aeruginosa* ΔKH-S1 mutant reveals multiple RNAs are under the influence of PNPase. Overexpression of *exsA* driven by a *lac* promoter restored the T3SS expression in the ΔKH-S1 mutant. On the chromosome, *exsA* was considered to be in the operon of *exsCEBA* (Hovey and Frank, 1995). However, a transcriptomic analysis revealed a gap between the RNAs of *exsA* and *exsCEB*, suggesting an independent promoter of *exsA* (Wurtzel et al., 2012). And a recent study demonstrated that an RNA helicases, DeaD is required for the translation of *exsA* (Intile et al., 2015). Based on these results, one possibility is that the *exsCEBA* transcript is subject to cleavage between *exsB* and *exsA* by endonuclease and PNPase, which is required for efficient translation of *exsA*. Other possible roles of PNPase on *exsA* expression might be that PNPase inhibits the translation of a negative regulator of *exsA* through its KH-S1 domains or PNPase degrades a sRNA that represses *exsA* expression.

In our study, we demonstrated that the levels of RsmY and RsmZ were increased in the ΔKH-S1 mutant, which was due to increased RNA stability. The degradation rates of RsmY and RsmZ in the ΔKH-S1 mutant were slower after rifampicin treatment. However, the RsmY/Z levels gradually dropped in the ΔKH-S1 mutant. These results suggest that PNPase might play an important role in the degradation process, while other RNAses are also involved in the degradation. The RNA EMSA demonstrated a direct binding between PNPase and RsmY/Z. Since structured RNAs are poor substrates of PNPase, we suspect that PNPase might bind to target RNAs, such as RsmY/Z through its KH-S1 domains. And RNA helicases are recruited to facilitate the degradation. It could be possible that under different environmental conditions, PNPase binds to different RNA helicases.

Overall, our results suggest that PNPase is involved in the regulation of multiple phenotypes, and an important mechanism is through the regulation of RsmY/Z stability. It would be interesting to screen the known sRNAs for PNPase targets. And the KH and S1 fragment of PNPase could be a useful tool to identify novel sRNAs as well as verify potential sRNAs. In addition, the mRNA targets of PNPase could also be identified by the KH-S1-RNA co-purification experiment.

## Author contributions

Conceived and designed the experiments: WW, SJ, RC, ZC. Performed the experiments: RC, YW, FZ, YJ, CL, XP, BX. Analyzed the data: RC, ZC, SJ, WW. Wrote the paper: RC, SJ, WW.

## Funding

This work was supported by National Science Foundation of China (31370168 to WW, and 31170128, 31370167 to SJ); National Basic Research Program of China (973 Program, 2012CB518700 and 2015DFG32500 to SJ) and Science and Technology Committee of Tianjin (13JCYBJC36700 to WW, 15JCZDJC33000 to SJ).

### Conflict of interest statement

The authors declare that the research was conducted in the absence of any commercial or financial relationships that could be construed as a potential conflict of interest.

## Supplementary material

Table S1 **Strains and plasmids used in this study**.

Table S2 **Primers used in real time PCR Strains and plasmids used in this study**.

Table S3 **Transcriptome analysis: differentially regulated genes**.

Figure S1 **(A)** Constructs of NTD-His. **(B)** Expression of NTD-His in the indicated strains. Wild type PAK and the ΔKH-S1 mutant containing a NTD-His in their chromosomes were grown for 12 h in LB. Samples from equivalent numbers of bacterial cells were loaded onto a SDS-PAGE gel and probed with an anti-His antibody.

Figure S2 **Secretion and expression of ExoS in indicated strains**. Bacteria were grown to an OD_600_ of 1.0 in LB with or without 5 mM EGTA. Lysates from equivalent bacterial cells were loaded onto SDS-PAGE gels and probed with the ExoS antibody.

Figure S3 **Relative RNA levels of *retS, ladS gacS, and gacA* in wild type PAK and the ΔKH-S1 mutant**. Bacteria were grown to an OD_600_ of 1.0. Total RNAs were purified and the relative mRNA levels were determined with real time PCR. ^*^*p* < 0.05 compared to wild type PAK by Student's *t*-test.

Figure S4 **Levels of Hfq mRNA in wild type PAK and the ΔKH-S1 mutant**. Bacteria were grown to an OD_600_ of 1.0. Total RNA from each strain was purified and analyzed with real time PCR.

Figure S5 **Growth of indicated strains**. The wild type PA14 containing pMMB67EH or the Δ*pnp* mutant containing pMMB67EH-*pnp* was grown in the presence of 100 μM IPTG for 16 h. Each bacterial culture was diluted into fresh LB to an OD_600_ of 0.03. The bacteria were then grown in the absence of presence of 100 μM IPTG with agitation. The bacteria concentration was determined by serial dilution and plating every hour.
