# Supplementary material for: Polynucleotide Phosphorylase Regulates Multiple Virulence Factors and the Stabilities of Small RNAs RsmY/Z in Pseudomonas aeruginosa
Source: Front Microbiol. 2016 Mar 2;7:247. doi: 10.3389/fmicb.2016.00247 (PMC4773659; doi:10.3389/fmicb.2016.00247)
Supplement: Table S1 — Strains and plasmids used in this study. [file Table1.DOC]

**Table S1 Strains and plasmids used in this study**

| **Strain** | **Description** | **Source (Reference)** |
| --- | --- | --- |
| ***P. aeruginosa*** |  |  |
| PAK | Wild-type *P. aeruginosa* strains | David Bradley |
| ΔKH-S1 | PAK with *pnp* (KH and S1) deletion | This study |
| ΔKH-S1/Tn7T-*pnp* | PAK ΔKH-S1 with *pnp* inserted on chromosome with mini-Tn7T insertion; Gm^r^ | This study |
| ΔKH | PAK ΔKH-S1 with *pnp* (KH deletion) inserted on chromosome with mini-Tn7T insertion; Gm^r^ | This study |
| ΔS1 | PAK ΔKH-S1 with *pnp* (S1 deletion) inserted on chromosome with mini-Tn7T insertion; Gm^r^ | This study |
| F639G | PAK ΔKH-S1 with *pnp* (F639 residue replaced with G) inserted on chromosome with mini-Tn7T insertion; Gm^r^ | This study |
| ΔKH-S1/Tn7T-Plac-*exsA* | PAK ΔKH-S1 with lac promoter -*exsA* inserted on chromosome with mini-Tn7T insertion; Gm^r^ | This study |
| Δ*rsmYZ* | PAK with *rsmY* and *rsmZ* deletion |  |
| ΔKH-S1Δ*rsmYZ* | PAK ΔKH-S1 with *rsmY* and *rsmZ* deletion | This study |
| **Plasmids** |  |  |
| pEX18Tc | Gene replacement vector; Tc^r^, *oriT*^+^, *sacB*^+^ | Jin lab |
| pET28b | Expression vector with T7 promoter; Kan^r^ | Jin lab |
| pUCP20 | Shuttle vector between *E. coli* and *P. aeruginosa;* Ap^r^ | Jin lab |
| pMMB67EH | Expression vector with *tac* promoter; Ap^r^ | Jin lab |
| pUC18T-mini-Tn7T-Gm | mini-Tn7 base vector from insertion into chromosome attTn7 site; Gm^r^ |  |
| pKW018 | *hcp*-1-FLAG on pUC18T-mini-Tn7T-Gm; Gm^r^ |  |
| pUC18T-mini-Tn7T-Gm-Plac-*exsA* | *lac* promoter driven *exsA* on pUC18T-mini-Tn7T-Gm; Gm^r^ |  |
| pCR001 | *pnp* (1755-2109) gene of PAK deletion on pEX18Tc; Tc^r^ | This study |
| pCR002 | *pnp* gene of PAK driven by its own promoter on pUC18T-mini-Tn7T-Gm; Gm^r^ | This study |
| pCR003 | *pnp* gene of PAK on pUCP20 driven by *lac* promoter; Ap^r^ | This study |
| pCR004 | *rsmY* promoter of PAK fused to promoterless *lacZ* on pDN19*lacZ*Ω; Sp^r^, Sm^r^, Tc^r^ | This study |
| pCR005 | *rsmZ* promoter of PAK fused to promoterless *lacZ* on pDN19*lacZ*Ω; Sp^r^, Sm^r^, Tc^r^ | This study |
| pCR006 | *pnp* gene with His-tag driven by *lac* promoter on pMMB67EH; Ap^r^ | This study |
| pCR007 | KH and S1 domain of *pnp* gene with His-tag driven by *lac* promoter on pMMB67EH; Ap^r^ | This study |
