# Supplementary material for: Polynucleotide Phosphorylase Regulates Multiple Virulence Factors and the Stabilities of Small RNAs RsmY/Z in Pseudomonas aeruginosa
Source: Front Microbiol. 2016 Mar 2;7:247. doi: 10.3389/fmicb.2016.00247 (PMC4773659; doi:10.3389/fmicb.2016.00247)
Supplement: Table S2 — Primers used in real time PCR Strains and plasmids used in this study. [file Table2.DOCX]

**Table S2. Primers used in real time PCR.**

| Primer | Sequence |
| --- | --- |
| exsA-F | 5’-GCTATGTCGTAAGTACCA-3’ |
| exsA-R | 5’-GAAGCCTTGTAGAAACTG-3’ |
|  |  |
| pilA-F | 5’-ATTGCCATTCCTCAGTAT-3’ |
| pilA-R | 5’-TCTTCAACGGTAGTCTTC-3’ |
|  |  |
| pilR-F | 5’-GATTCCCTCCGAACTGAT-3’ |
| pilR-R | 5’-CCTGCTTGTCTTCGATAG-3’ |
|  |  |
| hcp1-F | 5’-AGGACCTGTCGTTCACCAA-3’ |
| hcp1-R | 5’-ATAGTGCTTGCCGCTGGA-3’ |
|  |  |
| vgrG-F | 5’-GAGACCAGCTTCGACTTCATC-3’ |
| vgrG-R | 5’-CTTCTGCTCATGGCGGAAC-3’ |
|  |  |
| GacA-F | 5’-CCTGATGATCGCCAACTG-3’ |
| GacA-R | 5’-ATAGGTATTCACGGTCTTCG-3’ |
|  |  |
| GacS-F | 5’-ATCATCAACGAGATCCTC-3’ |
| GacS-R | 5’-GAGATTGAAAGGGAGGTT-3’ |
|  |  |
| LadS-F | 5’-GTGATGCTGATCTACAAC-3’ |
| LadS-R | 5’-CGAAGCGATATAGAGGAT-3’ |
|  |  |
| RetS-F | 5’-GATACTCGACATCTCCAA-3’ |
| RetS-R | 5’-GAAGATATCCAGGCAGTC-3’ |
|  |  |
| RsmA-F | 5’-GAAGGAAGTCGCCGTACA-3’ |
| RsmA-R | 5’-TAATGGTTTGGCTCTTGATCTTTC-3’ |
|  |  |
| RpsL-F | 5’-CAAAACTGCCCGCAACGT-3’ |
| RpsL-R | 5’-TTTCGGCGTGGTGGTGTAT-3’ |
|  |  |
| PA1805-F | 5’-ATATCAGTCTCAATGAAGTC-3’ |
| PA1805-R | 5’-CATGGATGGATCGAAATC-3’ |
|  |  |
| RsmY-F | 5’-TCAGGACATTGCGCAGGAA-3’ |
| RsmY-R | 5’-TTTGCAGACCTCTATCCTGACATC-3’ |
|  |  |
| RsmZ-F | 5’-GGAACACGCAACCCCGAAGG-3’ |
| RsmZ-R | 5’-CCGCCCACTCTTCAGTCCCT-3’ |
|  |  |
| proC-F | 5’-CAGGCCGGGCAGTTGCTGTC-3’ |
| proC-R | 5’-GGTCAGGCGCGAGGCTGTCT-3’ |
