# Supplementary material for: Polynucleotide Phosphorylase Regulates Multiple Virulence Factors and the Stabilities of Small RNAs RsmY/Z in Pseudomonas aeruginosa
Source: Front Microbiol. 2016 Mar 2;7:247. doi: 10.3389/fmicb.2016.00247 (PMC4773659; doi:10.3389/fmicb.2016.00247)
Supplement: Table S3 — Transcriptome analysis: differentially regulated genes. [file Table3.DOC]

**Table S3**. Transcriptome analysis: differentially regulated genes

| Locus | Name | Product | Fold changes  (ΔKH-S1/WT) | *P* value |
| --- | --- | --- | --- | --- |
| PA0020 | PA0020 | hypothetical protein | 0.4566 | 0.015953 |
| PA0044 | *exoT* | exoenzyme T | 0.15991 | 1.02E-05 |
| PA0045 | PA0045 | hypothetical protein | 0.47169 | 0.019736 |
| PA0086 | PA0086 | hypothetical protein | 3.7406 | 0.037179 |
| PA0087 | PA0087 | hypothetical protein | 4.46793 | 0.019493 |
| PA0090 | *clpV1* | ClpV1 protein | 3.51433 | 0.047076 |
| PA0091 | *vgrG1* | VgrG protein | 3.50151 | 0.049148 |
| PA0105 | *coxB* | cytochrome C oxidase subunit II | 4.60462 | 0.012635 |
| PA0160 | PA0160 | hypothetical protein | 0.33361 | 0.028123 |
| PA0164 | PA0164 | gamma-glutamyltranspeptidase | 3.49271 | 0.04958 |
| PA0165 | PA0165 | hypothetical protein | 0.28181 | 0.000817 |
| PA0169 | PA0169 | hypothetical protein | 4.75284 | 0.010449 |
| PA0170 | PA0170 | hypothetical protein | 4.29434 | 0.022174 |
| PA0171 | PA0171 | hypothetical protein | 3.61579 | 0.049371 |
| PA0172 | PA0172 | hypothetical protein | 8.35016 | 0.000242 |
| PA0284 | PA0284 | hypothetical protein | 0.24008 | 0.001222 |
| PA0291 | *oprE* | anaerobically-induced outer membrane porin OprE | 0.3351 | 0.002411 |
| PA0359 | PA0359 | hypothetical protein | 0.40456 | 0.008428 |
| PA0391 | PA0391 | hypothetical protein | 0.37878 | 0.005332 |
| PA0396 | *pilU* | twitching motility protein PilU | 0.3314 | 0.002268 |
| PA0411 | *pilJ* | twitching motility protein PilJ | 0.43396 | 0.011866 |
| PA0412 | *pilK* | methyltransferase PilK | 0.31908 | 0.001878 |
| PA0413 | *chpA* | chemotactic signal transduction system protein | 0.49434 | 0.024512 |
| PA0414 | *chpB* | methylesterase | 0.45507 | 0.016317 |
| PA0415 | *chpC* | chemotaxis protein | 0.52074 | 0.036402 |
| PA0416 | *chpD* | transcriptional regulator | 0.54752 | 0.049661 |
| PA0446 | PA0446 | hypothetical protein | 4.53731 | 0.011848 |
| PA0447 | *gcdH* | glutaryl-CoA dehydrogenase | 4.12849 | 0.019664 |
| PA0449 | PA0449 | hypothetical protein | 0.52299 | 0.034395 |
| PA0492 | PA0492 | hypothetical protein | 0.22973 | 0.000209 |
| PA0493 | PA0493 | hypothetical protein | 0.44684 | 0.02049 |
| PA0494 | PA0494 | acetyl-CoA carboxylase biotin carboxylase subunit | 0.38266 | 0.005873 |
| PA0495 | PA0495 | hypothetical protein | 0.45641 | 0.016679 |
| PA0496 | PA0496 | hypothetical protein | 0.54056 | 0.041209 |
| PA0506 | PA0506 | acyl-CoA dehydrogenase | 0.53373 | 0.037144 |
| PA0510 | *nirN* | NirN | 0.5224 | 0.035426 |
| PA0511 | *nirJ* | heme d1 biosynthesis protein NirJ | 0.5452 | 0.044781 |
| PA0526 | PA0526 | hypothetical protein | 0.49185 | 0.039419 |
| PA0538 | *dsbB* | disulfide bond formation protein | 0.49563 | 0.028494 |
| PA0546 | *metK* | S-adenosylmethionine synthetase | 0.56147 | 0.047848 |
| PA0602 | PA0602 | ABC transporter | 0.48543 | 0.022533 |
| PA0635 | PA0635 | hypothetical protein | 0.39346 | 0.038112 |
| PA0652 | *vfr* | cAMP-regulatory protein | 0.51701 | 0.031522 |
| PA0654 | *speD* | S-adenosylmethionine decarboxylase | 0.44256 | 0.013775 |
| PA0668.1 | PA0668.1 | 16S ribosomal RNA | 27.0399 | 1.03E-08 |
| PA0668.4 | PA0668.4 | 23S ribosomal RNA | 23.8394 | 3.20E-08 |
| PA0674 | *vreA* | VreA protein | 5.65179 | 0.012139 |
| PA0696 | PA0696 | hypothetical protein | 3.70166 | 0.048252 |
| PA0713 | PA0713 | hypothetical protein | 0.3492 | 0.004904 |
| PA0782 | *putA* | bifunctional proline dehydrogenase Fpyrroline-5-carboxylate dehydrogenase | 0.1666 | 1.39E-05 |
| PA0783 | *putP* | Sodium Fproline symporter PutP | 0.2431 | 0.00026 |
| PA0788a | PA0788a | hypothetical protein | 0.46376 | 0.047081 |
| PA0805 | PA0805 | hypothetical protein | 5.60742 | 0.003101 |
| PA0839 | PA0839 | transcriptional regulator | 0.49508 | 0.029107 |
| PA0844 | *plcH* | hemolytic phospholipase C | 4.01993 | 0.032862 |
| PA0852 | *cbpD* | chitin-binding protein CbpD | 0.24936 | 0.000391 |
| PA0892 | *aotP* | Arginine Fornithine transporter AotP | 0.5443 | 0.042006 |
| PA0897 | *aruG* | Arginine Fornithine succinyltransferase AII subunit | 0.56416 | 0.04959 |
| PA0898 | *aruD* | succinylglutamic semialdehyde dehydrogenase | 0.45366 | 0.01581 |
| PA0899 | *aruB* | succinylarginine dihydrolase | 0.49925 | 0.026289 |
| PA0921 | PA0921 | hypothetical protein | 4.07141 | 0.022748 |
| PA0957 | PA0957 | hypothetical protein | 3.94301 | 0.028059 |
| PA0993 | *cupC2* | chaperone CupC2 | 6.94118 | 0.002442 |
| PA1228 | PA1228 | hypothetical protein | 0.38217 | 0.008087 |
| PA1393 | *cysC* | adenosine 5-phosphosulfate APS kinase | 3.8734 | 0.036543 |
| PA1395 | PA1395 | hypothetical protein | 4.16314 | 0.040976 |
| PA1396 | PA1396 | two-component sensor | 4.01664 | 0.028561 |
| PA1421 | *gbuA* | guanidinobutyrase | 0.46365 | 0.021927 |
| PA1498 | *pykF* | pyruvate kinase | 3.71875 | 0.0483 |
| PA1515 | *alc* | allantoicase | 3.81367 | 0.033598 |
| PA1523 | *xdhB* | xanthine dehydrogenase | 3.51451 | 0.049057 |
| PA1553 | *ccoO1* | cbb3-type cytochrome C oxidase subunit II | 0.54403 | 0.04121 |
| PA1670 | *stp1* | Serine Fthreonine phosphoprotein phosphatase Stp1 | 4.24523 | 0.036998 |
| PA1688 | PA1688 | hypothetical protein | 5.41613 | 0.004218 |
| PA1691 | *pscT* | translocation protein in type III secretion | 0.53217 | 0.049187 |
| PA1692 | PA1692 | probable translocation protein in type III secretion | 0.25461 | 0.00206 |
| PA1693 | *pscR* | type III secretion system protein | 0.20722 | 0.000117 |
| PA1694 | *pscQ* | type III secretion system protein | 0.22625 | 0.000302 |
| PA1695 | *pscP* | translocation protein in type III secretion | 0.23682 | 0.000338 |
| PA1696 | *pscO* | translocation protein in type III secretion | 0.21692 | 0.00026 |
| PA1697 | *pscO* |  | 0.32053 | 0.002085 |
| PA1698 | *popN* | type III secretion outer membrane protein PopN | 0.29444 | 0.001205 |
| PA1699 | *popN* |  | 0.23062 | 0.00051 |
| PA1700 | PA1700 | hypothetical protein | 0.26403 | 0.001281 |
| PA1701 | PA1701 | hypothetical protein | 0.25247 | 0.001333 |
| PA1703 | *pcrD* | type III secretory apparatus protein PcrD | 0.35261 | 0.003461 |
| PA1704 | *pcrR* | transcriptional regulator PcrR | 0.32075 | 0.006457 |
| PA1705 | *pcrG* | regulator in type III secretion | 0.40412 | 0.010673 |
| PA1706 | *pcrV* | type III secretion protein PcrV | 0.3169 | 0.001794 |
| PA1707 | *pcrH* | regulatory protein PcrH | 0.15522 | 1.23E-05 |
| PA1708 | *popB* | translocator protein PopB | 0.21644 | 0.00011 |
| PA1709 | *popD* | translocator outer membrane protein PopD | 0.1881 | 3.83E-05 |
| PA1710 | *exsC* | exoenzyme S synthesis protein C | 0.48008 | 0.022071 |
| PA1711 | *exsE* | ExsE protein | 0.46633 | 0.021247 |
| PA1713 | *exsA* | transcriptional regulator ExsA | 0.38768 | 0.006181 |
| PA1714 | *exsD* | ExsD protein | 0.34449 | 0.003011 |
| PA1715 | *pscB* | type III export apparatus protein | 0.24135 | 0.000354 |
| PA1716 | *pscC* | type III secretion outer membrane protein PscC | 0.31388 | 0.001609 |
| PA1717 | *pscD* | type III export protein PscD | 0.27541 | 0.000721 |
| PA1718 | *pscE* | type III export protein PscE | 0.07945 | 5.95E-06 |
| PA1719 | *pscF* | type III export protein PscF | 0.11164 | 7.59E-07 |
| PA1720 | *pscG* | type III export protein PscG | 0.11603 | 1.24E-06 |
| PA1721 | *pscH* | type III export protein PscH | 0.15169 | 1.26E-05 |
| PA1722 | *pscI* | type III export protein PscI | 0.19506 | 6.60E-05 |
| PA1723 | *pscJ* | type III export protein PscJ | 0.24424 | 0.000288 |
| PA1724 | *pscK* | type III export protein PscK | 0.21932 | 0.000216 |
| PA1725 | *pscL* | type III secretion system protein | 0.28892 | 0.001065 |
| PA1837 | PA1837 | hypothetical protein | 0.40818 | 0.012996 |
| PA1867 | *xphA* | XphA protein | 0.19115 | 0.003833 |
| PA1868 | *xqhA* | secretion protein XqhA | 0.1812 | 4.03E-05 |
| PA1869 | PA1869 | acyl carrier protein | 0.1861 | 0.000191 |
| PA1913 | PA1913 | hypothetical protein | 6.52485 | 0.001719 |
| PA1914 | PA1914 | hypothetical protein | 3.72901 | 0.044365 |
| PA1974 | PA1974 | hypothetical protein | 7.55208 | 0.003989 |
| PA2006 | PA2006 | major facilitator superfamily MFS transporter | 4.9069 | 0.008904 |
| PA2007 | *maiA* | maleylacetoacetate isomerase | 3.61134 | 0.04265 |
| PA2021 | PA2021 | hypothetical protein | None | 0.001996 |
| PA2031 | PA2031 | hypothetical protein | 0.28608 | 0.016273 |
| PA2044 | PA2044 | hypothetical protein | 0.43014 | 0.011679 |
| PA2109 | PA2109 | hypothetical protein | 6.20324 | 0.003783 |
| PA2110 | PA2110 | hypothetical protein | 4.72271 | 0.011924 |
| PA2111 | PA2111 | hypothetical protein | 3.77755 | 0.03743 |
| PA2112 | PA2112 | LamB YcsF family protein | 4.26835 | 0.019996 |
| PA2113 | *opdO* | pyroglutatmate porin OpdO | 3.63886 | 0.042027 |
| PA2114 | PA2114 | major facilitator superfamily MFS transporter | 3.9403 | 0.026389 |
| PA2157 | PA2157 | hypothetical protein | 4.216 | 0.042332 |
| PA2158 | PA2158 | alcohol dehydrogenase | 6.91304 | 0.006944 |
| PA2168 | PA2168 | hypothetical protein | 18.4706 | 0.007447 |
| PA2172 | PA2172 | hypothetical protein | 5.74074 | 0.017862 |
| PA2187 | PA2187 | hypothetical protein | 0.26037 | 0.005074 |
| PA2189 | PA2189 | hypothetical protein | 0.32413 | 0.00267 |
| PA2191 | *exoY* | adenylate cyclase | 0.29325 | 0.001068 |
| PA2197 | PA2197 | hypothetical protein | 0.5408 | 0.045942 |
| PA2240 | *pslJ* | protein PslJ | 3.90287 | 0.030241 |
| PA2308 | PA2308 | ABC transporter ATP-binding protein | 6.44681 | 0.038199 |
| PA2312 | PA2312 | transcriptional regulator | 0.4 | 0.024761 |
| PA2340 | PA2340 | binding-protein-dependent maltose Fmannitol transport protein | 4.5 | 0.047336 |
| PA2352 | PA2352 | glycerophosphoryl diester phosphodiesterase | 0.3085 | 0.001608 |
| PA2361 | PA2361 | hypothetical protein | 3.84082 | 0.036642 |
| PA2370 | PA2370 | hypothetical protein | 7.76471 | 0.019615 |
| PA2382 | *lldA* | L-lactate dehydrogenase | 5.74491 | 0.003904 |
| PA2436 | PA2436 | hypothetical protein | 0.43314 | 0.013471 |
| PA2453 | PA2453 | hypothetical protein | 0.2034 | 0.0001 |
| PA2538 | PA2538 | hypothetical protein | 4.08365 | 0.041103 |
| PA2550 | PA2550 | acyl-CoA dehydrogenase | 0.52758 | 0.036703 |
| PA2560 | PA2560 | hypothetical protein | 7.46357 | 0.000984 |
| PA2571 | PA2571 | two-component sensor | 3.72148 | 0.043179 |
| PA2596 | PA2596 | hypothetical protein | 4.49505 | 0.028043 |
| PA2669 | PA2669 | hypothetical protein | 4.27725 | 0.039155 |
| PA2687 | *pfeS* | two-component sensor PfeS | 4.32396 | 0.018636 |
| PA2697 | PA2697 | hypothetical protein | None | 0.034783 |
| PA2739 | *pheT* | phenylalanyl-tRNA synthetase subunit beta | 0.54223 | 0.040033 |
| PA2746 | PA2746 | hypothetical protein | 4.46809 | 0.046668 |
| PA2751 | PA2751 | hypothetical protein | 5.86254 | 0.006454 |
| PA2755a | PA2755a | hypothetical protein | 5.63206 | 0.004634 |
| PA2774 | PA2774 | hypothetical protein | 4.2061 | 0.022362 |
| PA2775 | PA2775 | hypothetical protein | 3.71314 | 0.042742 |
| PA2782 | PA2782 | hypothetical protein | 0.09743 | 1.19E-06 |
| PA2783 | PA2783 | hypothetical protein | 0.07925 | 3.82E-08 |
| PA2793 | PA2793 | hypothetical protein | 4.53566 | 0.013442 |
| PA2810 | *copS* | two-component sensor CopS | 5.55878 | 0.003611 |
| PA2855 | PA2855 | hypothetical protein | 4.17564 | 0.026011 |
| PA2860 | PA2860 | hypothetical protein | 4.03804 | 0.022823 |
| PA2874 | PA2874 | hypothetical protein | 0.43902 | 0.015845 |
| PA2916 | PA2916 | hypothetical protein | 5.09135 | 0.03349 |
| PA2938 | PA2938 | transporter | 4.88506 | 0.012158 |
| PA2958.1 | *rgsA* | RgsA | 5.21614 | 0.005172 |
| PA3001 | PA3001 | glyceraldehyde-3-phosphate dehydrogenase | 0.53915 | 0.038782 |
| PA3006 | *psrA* | transcriptional regulator PsrA | 0.55582 | 0.045559 |
| PA3057 | PA3057 | hypothetical protein | 0.45795 | 0.042332 |
| PA3059 | *pelF* | protein PelF | 5.52508 | 0.004936 |
| PA3060 | *pelE* | protein PelE | 4.03186 | 0.034716 |
| PA3063 | *pelB* | protein PelB | 5.90203 | 0.002977 |
| PA3064 | *pelA* | PelA protein | 4.58904 | 0.013927 |
| PA3069 | PA3069 | hypothetical protein | 3.9399 | 0.034857 |
| PA3091 | PA3091 | hypothetical protein | 0.38788 | 0.00632 |
| PA3099 | *xcpV* | general secretion pathway protein I | 0.49631 | 0.040111 |
| PA3236 | PA3236 | glycine betaine-binding protein | 5.58776 | 0.004469 |
| PA3274 | PA3274 | hypothetical protein | 0.35068 | 0.012139 |
| PA3340 | PA3340 | hypothetical protein | 4.22385 | 0.018741 |
| PA3375 | PA3375 | ABC transporter ATP-binding protein | 6.22581 | 0.03354 |
| PA3384 | *phnC* | phosphonate ABC transporter ATP-binding protein | 3.99094 | 0.047679 |
| PA3385 | *amrZ* | alginate and motility regulator Z | 0.55382 | 0.045584 |
| PA3446 | PA3446 | NADH-dependent FMN reductase | 0.46347 | 0.021631 |
| PA3450 | PA3450 | antioxidant protein | 0.49981 | 0.027791 |
| PA3467 | PA3467 | major facilitator superfamily MFS transporter | 6.80952 | 0.003101 |
| PA3479 | *rhlA* | rhamnosyltransferase subunit A | 5.23308 | 0.029464 |
| PA3517 | PA3517 | adenylosuccinate lyase | 3.95611 | 0.039112 |
| PA3609 | *potC* | polyamine transporter PotC | 0.29219 | 0.002539 |
| PA3610 | *potD* | polyamine transporter PotD | 0.41 | 0.010477 |
| PA3635 | *eno* | phosphopyruvate hydratase | 0.51391 | 0.03031 |
| PA3641 | PA3641 | amino acid permease | 0.41995 | 0.009898 |
| PA3656 | *rpsB* | 30S ribosomal protein S2 | 0.45795 | 0.016191 |
| PA3713 | *spdH* | spermidine dehydrogenase SpdH | 0.24319 | 0.000288 |
| PA3722 | PA3722 | hypothetical protein | 5.13555 | 0.007276 |
| PA3742 | *rplS* | 50S ribosomal protein L19 | 0.30389 | 0.001278 |
| PA3789 | PA3789 | hypothetical protein | 0.5549 | 0.047997 |
| PA3790 | *oprC* | copper transport outer membrane porin OprC | 0.56275 | 0.04902 |
| PA3841 | *exoS* | exoenzyme S | 0.14158 | 3.72E-06 |
| PA3842 | PA3842 | chaperone | 0.20523 | 0.000104 |
| PA3877 | *narK1* | nitrite extrusion protein 1 | 0.32403 | 0.001933 |
| PA3906 | PA3906 | hypothetical protein | 22.0526 | 0.004066 |
| PA3914 | *moeA1* | molybdenum cofactor biosynthetic protein A1 | 0.30269 | 0.001373 |
| PA3915 | *moaB1* | molybdopterin biosynthetic protein B1 | 0.16761 | 1.66E-05 |
| PA3916 | *moaE* | molybdopterin converting factor large subunit | 0.52807 | 0.040777 |
| PA3918 | *moaC* | molybdenum cofactor biosynthesis protein MoaC | 0.43722 | 0.013402 |
| PA3931 | PA3931 | hypothetical protein | 0.27764 | 0.001011 |
| PA3940 | PA3940 | DNA binding protein | 0.55132 | 0.044572 |
| PA3956 | PA3956 | hypothetical protein | 0.51946 | 0.039863 |
| PA3973 | PA3973 | transcriptional regulator | 0.54808 | 0.044623 |
| PA4031 | *ppa* | inorganic pyrophosphatase | 0.52609 | 0.034926 |
| PA4032 | PA4032 | two-component response regulator | 3.74813 | 0.035736 |
| PA4060 | PA4060 | hypothetical protein | None | 0.014079 |
| PA4085 | *cupB2* | chaperone CupB2 | 5.66434 | 0.010598 |
| PA4108 | PA4108 | cyclic di-GMP phosphodiesterase | 0.39036 | 0.0067 |
| PA4138 | *tyrS* | tyrosyl-tRNA synthetase | 0.53088 | 0.04007 |
| PA4179 | PA4179 | porin | 0.37031 | 0.005481 |
| PA4195 | PA4195 | ABC transporter | 0.39687 | 0.008614 |
| PA4242 | *rpmJ* | 50S ribosomal protein L36 | 0.51979 | 0.042428 |
| PA4247 | *rplR* | 50S ribosomal protein L18 | 0.53954 | 0.039484 |
| PA4261 | *rplW* | 50S ribosomal protein L23 | 0.56268 | 0.048952 |
| PA4271 | *rplL* | 50S ribosomal protein L7%2FL12 | 0.47724 | 0.020549 |
| PA4272 | *rplJ* | 50S ribosomal protein L10 | 0.52475 | 0.033931 |
| PA4272.1 | *rplJ* | P27 | 0.43052 | 0.011811 |
| PA4273 | *rplA* | 50S ribosomal protein L1 | 0.51442 | 0.030486 |
| PA4280.2 | PA4280.2 | 23S ribosomal RNA | 6.97619 | 0.002615 |
| PA4280.5 | PA4280.5 | 16S ribosomal RNA | 17.2315 | 5.63E-07 |
| PA4302 | *tadA* | ATPase TadA | 5.12613 | 0.012623 |
| PA4307 | *pctC* | chemotactic transducer PctC | 0.55193 | 0.044301 |
| PA4310 | *pctB* | chemotactic transducer PctB | 0.36214 | 0.004035 |
| PA4333 | PA4333 | fumarase | 0.43801 | 0.012618 |
| PA4343 | PA4343 | major facilitator superfamily MFS transporter | 5.10345 | 0.03349 |
| PA4354 | PA4354 | hypothetical protein | 12.4907 | 2.36E-05 |
| PA4360a | PA4360a | hypothetical protein | 8.01613 | 0.039696 |
| PA4369 | PA4369 | hypothetical protein | 0.41718 | 0.011542 |
| PA4396 | PA4396 | two-component response regulator | 0.53996 | 0.041548 |
| PA4429 | PA4429 | cytochrome C1 | 0.54017 | 0.039321 |
| PA4432 | *rpsI* | 30S ribosomal protein S9 | 0.47956 | 0.021128 |
| PA4523 | PA4523 | hypothetical protein | 0.4009 | 0.007467 |
| PA4525 | *pilA* | type 4 fimbrial PilA | 0.51187 | 0.030077 |
| PA4527 | *pilC* |  | 0.54672 | 0.041833 |
| PA4547 | *pilR* | two-component response regulator PilR | 0.46878 | 0.019024 |
| PA4550 | *fimU* | type 4 fimbrial biogenesis protein FimU | 0.39812 | 0.007478 |
| PA4551 | *pilV* | type 4 fimbrial biogenesis protein PilV | 0.35586 | 0.003935 |
| PA4552 | *pilW* | type 4 fimbrial biogenesis protein PilW | 0.36734 | 0.004521 |
| PA4553 | *pilX* | type 4 fimbrial biogenesis protein PilX | 0.43412 | 0.012812 |
| PA4554 | *pilY1* | type 4 fimbrial biogenesis protein PilY1 | 0.40549 | 0.008012 |
| PA4555 | *pilY2* | type 4 fimbrial biogenesis protein PilY2 | 0.37192 | 0.005566 |
| PA4556 | *pilE* | type 4 fimbrial biogenesis protein PilE | 0.35542 | 0.003868 |
| PA4567 | *rpmA* | 50S ribosomal protein L27 | 0.50591 | 0.028429 |
| PA4578 | PA4578 | hypothetical protein | 0.42826 | 0.011148 |
| PA4582 | PA4582 | hypothetical protein | 9.78522 | 6.58E-05 |
| PA4583 | PA4583 | hypothetical protein | 10.4903 | 3.75E-05 |
| PA4584 | PA4584 | hypothetical protein | 7.59627 | 0.000459 |
| PA4585 | *rtcA* | RNA terminal-phosphate cyclase | 5.23021 | 0.005891 |
| PA4602 | *glyA3* | serine hydroxymethyltransferase | 0.54887 | 0.042754 |
| PA4610 | PA4610 | hypothetical protein | 0.50318 | 0.030175 |
| PA4616 | PA4616 | c4-dicarboxylate-binding protein | 0.55594 | 0.047221 |
| PA4624 | PA4624 | hypothetical protein | 3.62609 | 0.042097 |
| PA4625 | PA4625 | hypothetical protein | 5.36266 | 0.00435 |
| PA4635 | PA4635 | hypothetical protein | 4.63415 | 0.019323 |
| PA4669 | *ipk* | 4-diphosphocytidyl-2-C-methyl-D-erythritol kinase | 5.03971 | 0.006114 |
| PA4671 | PA4671 | 50S ribosomal protein L25 | 0.5204 | 0.032446 |
| PA4690.2 | PA4690.2 | 23S ribosomal RNA | 9.97297 | 5.12E-05 |
| PA4697 | PA4697 | hypothetical protein | 0.50542 | 0.029002 |
| PA4704.1 | *prrF1* | regulatory RNA PrrF1 | 0.3944 | 0.039955 |
| PA4738 | PA4738 | hypothetical protein | 3.84047 | 0.034943 |
| PA4739 | PA4739 | hypothetical protein | 4.36491 | 0.015865 |
| PA4762 | *grpE* | heat shock protein GrpE | 0.48486 | 0.022563 |
| PA4770 | *lldP* | L-lactate permease | 13.476 | 4.97E-06 |
| PA4771 | *lldD* | L-lactate dehydrogenase | 11.8474 | 1.47E-05 |
| PA4772 | PA4772 | ferredoxin | 15.1629 | 1.79E-06 |
| PA4813 | *lipC* | lipase LipC | 4.9395 | 0.017165 |
| PA4853 | *fis* | Fis family transcriptional regulator | 0.40114 | 0.008405 |
| PA4858 | PA4858 | hypothetical protein | 3.68274 | 0.043762 |
| PA4913 | PA4913 | ABC transporter | 3.53414 | 0.048594 |
| PA4932 | *rplI* | 50S ribosomal protein L9 | 0.42164 | 0.010152 |
| PA4933 | PA4933 | hypothetical protein | 0.50463 | 0.027585 |
| PA4934 | *rpsR* | 30S ribosomal protein S18 | 0.41552 | 0.009531 |
| PA4935 | *rpsF* | 30S ribosomal protein S6 | 0.49012 | 0.02377 |
| PA4985 | PA4985 | hypothetical protein | 4.99816 | 0.008351 |
| PA4986 | PA4986 | oxidoreductase | 3.84519 | 0.031113 |
| PA5040 | *pilQ* | type 4 fimbrial biogenesis outer membrane protein PilQ | 0.35804 | 0.003676 |
| PA5041 | *pilP* | type 4 fimbrial biogenesis protein PilP | 0.38642 | 0.00605 |
| PA5042 | *pilO* | type 4 fimbrial biogenesis protein PilO | 0.46202 | 0.017119 |
| PA5043 | *pilN* | type 4 fimbrial biogenesis protein PilN | 0.51759 | 0.031694 |
| PA5044 | *pilM* | type 4 fimbrial biogenesis protein PilM | 0.50816 | 0.028528 |
| PA5087 | PA5087 | hypothetical protein | 3.97289 | 0.046096 |
| PA5139 | PA5139 | hypothetical protein | 0.29507 | 0.001131 |
| PA5172 | *arcB* | ornithine carbamoyltransferase | 0.35171 | 0.003296 |
| PA5173 | *arcC* | carbamate kinase | 0.22737 | 0.000158 |
| PA5286 | PA5286 | hypothetical protein | 3.53814 | 0.047278 |
| PA5369.2 | PA5369.2 | 23S ribosomal RNA | 9.97297 | 5.12E-05 |
| PA5375 | *betT1* | choline transporter BetT | 4.92258 | 0.008936 |
| PA5396 | PA5396 | hypothetical protein | 5.542 | 0.003908 |
| PA5397 | PA5397 | hypothetical protein | 6.08784 | 0.003368 |
| PA5398 | *dgcA* | dimethylglycine catabolism protein DgcA | 5.43544 | 0.004366 |
| PA5403 | PA5403 | transcriptional regulator | 4.00494 | 0.036503 |
| PA5406 | PA5406 | hypothetical protein | 0.35468 | 0.006046 |
| PA5410 | *gbcA* | protein GbcA | 9.73176 | 9.23E-05 |
| PA5411 | *gbcB* | protein GbcB | 4 | 0.027852 |
| PA5416 | *soxB* | sarcosine oxidase subunit beta | 8.93953 | 0.000257 |
| PA5418 | *soxA* | sarcosine oxidase subunit alpha | 6.33151 | 0.001719 |
| PA5419 | *soxG* | sarcosine oxidase subunit gamma | 4.07031 | 0.044849 |
| PA5420 | *purU2* | formyltetrahydrofolate deformylase | 5.60894 | 0.006192 |
| PA5421 | *fdhA* | glutathione-independent formaldehyde dehydrogenase | 3.85963 | 0.035476 |
| PA5472 | PA5472 | hypothetical protein | 0.44634 | 0.015304 |
| PA5559 | *atpE* | ATP synthase F0F1 subunit C | 0.50185 | 0.027275 |
