# Supplementary figures and images for: Polynucleotide Phosphorylase Regulates Multiple Virulence Factors and the Stabilities of Small RNAs RsmY/Z in Pseudomonas aeruginosa
Source: Front Microbiol. 2016 Mar 2;7:247. doi: 10.3389/fmicb.2016.00247 (PMC4773659; doi:10.3389/fmicb.2016.00247)

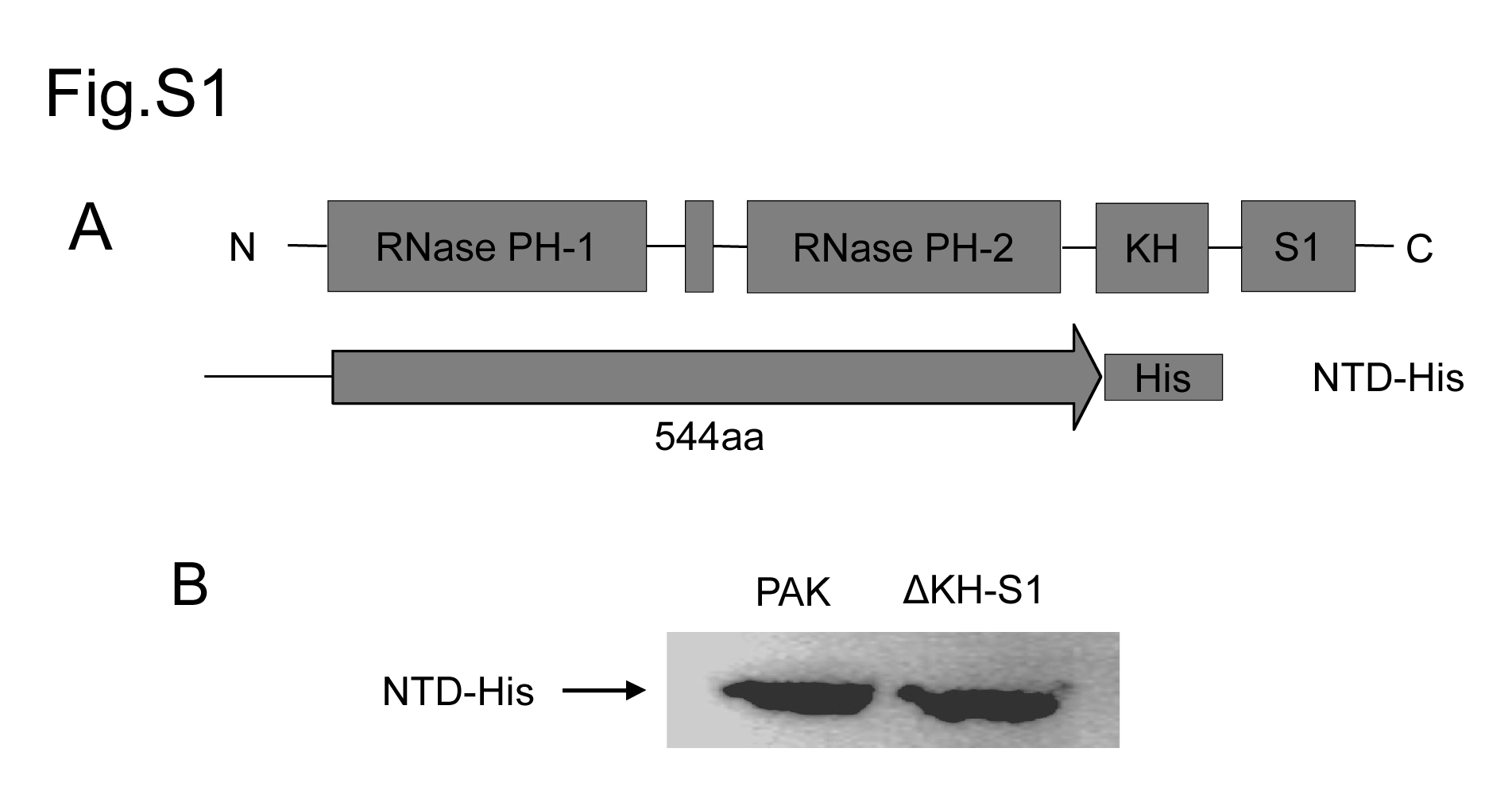

Supplement: Figure S1 — (A) Constructs of NTD-His. (B) Expression of NTD-His in the indicated strains. Wild type PAK and the ΔKH-S1 mutant containing a NTD-His in their chromosomes were grown for 12 h in LB. Samples from equivalent numbers of bacterial cells were loaded onto a SDS-PAGE gel and probed with an anti-His antibody. [file Image1.TIF]

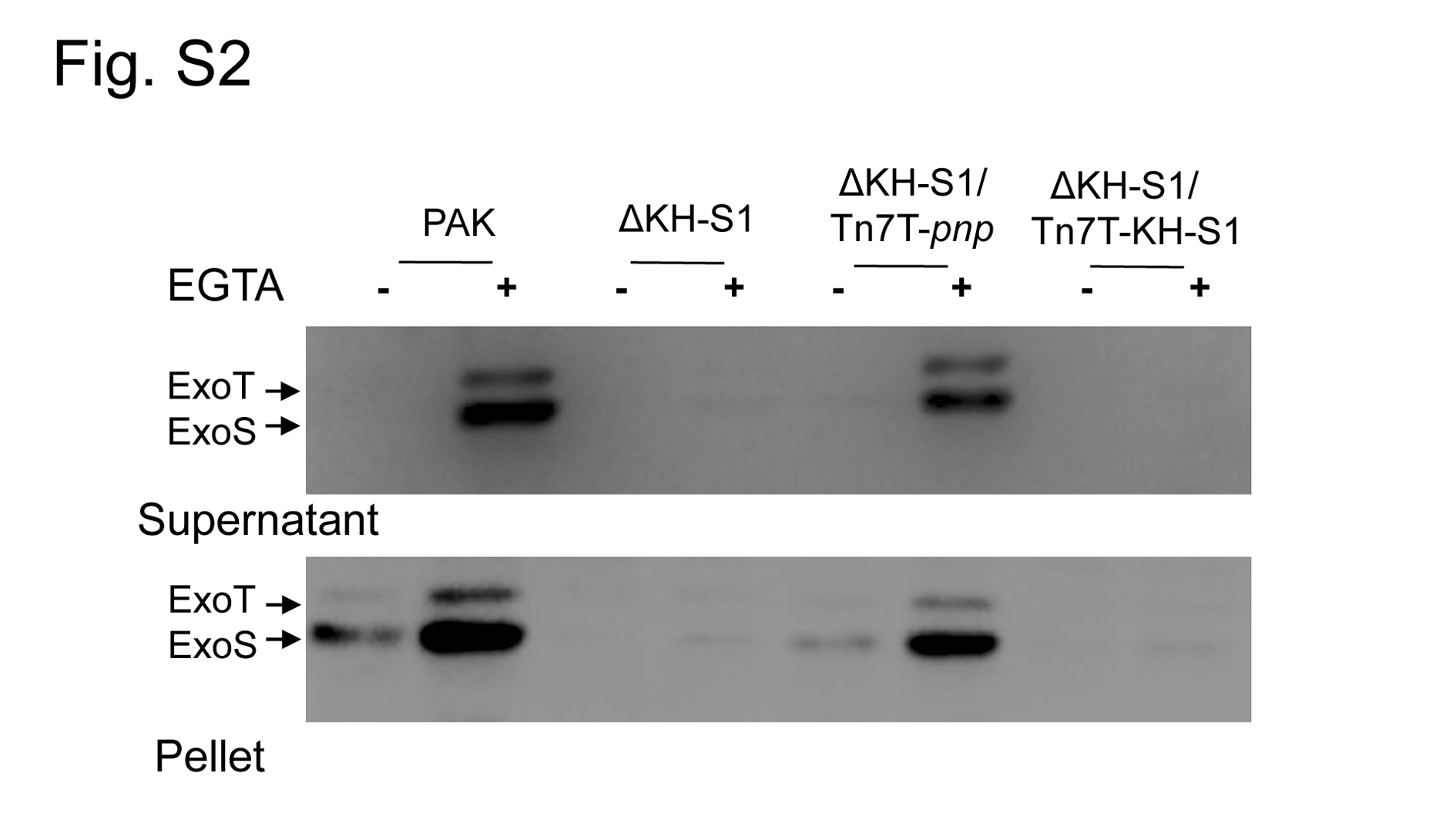

Supplement: Figure S2 — Secretion and expression of ExoS in indicated strains. Bacteria were grown to an OD600 of 1.0 in LB with or without 5 mM EGTA. Lysates from equivalent bacterial cells were loaded onto SDS-PAGE gels and probed with the ExoS antibody. [file Image2.TIF]

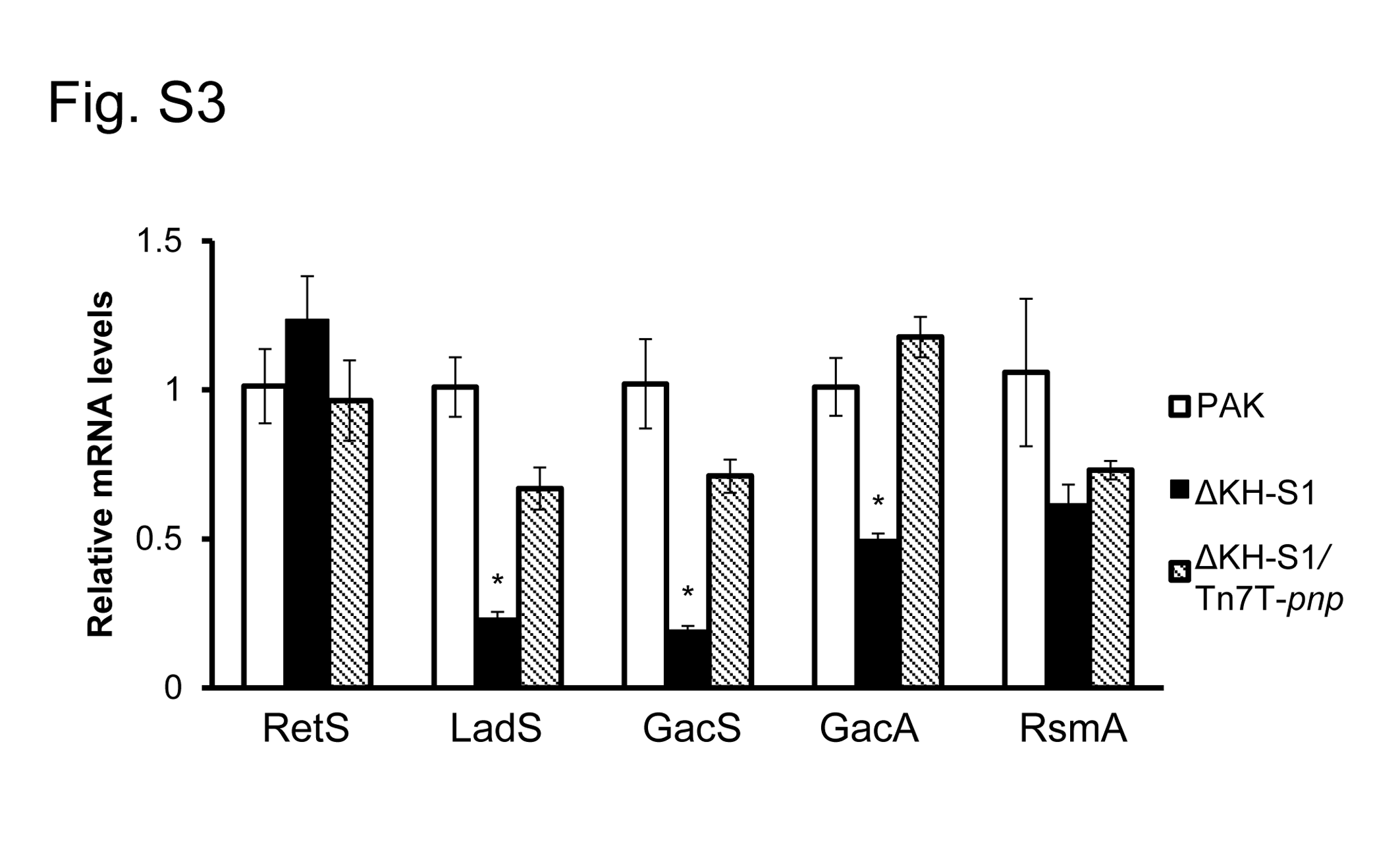

Supplement: Figure S3 — Relative RNA levels of retS, ladS gacS, and gacA in wild type PAK and the ΔKH-S1 mutant. Bacteria were grown to an OD600 of 1.0. Total RNAs were purified and the relative mRNA levels were determined with real time PCR. *p < 0.05 compared to wild type PAK by Student's t-test. [file Image3.TIF]

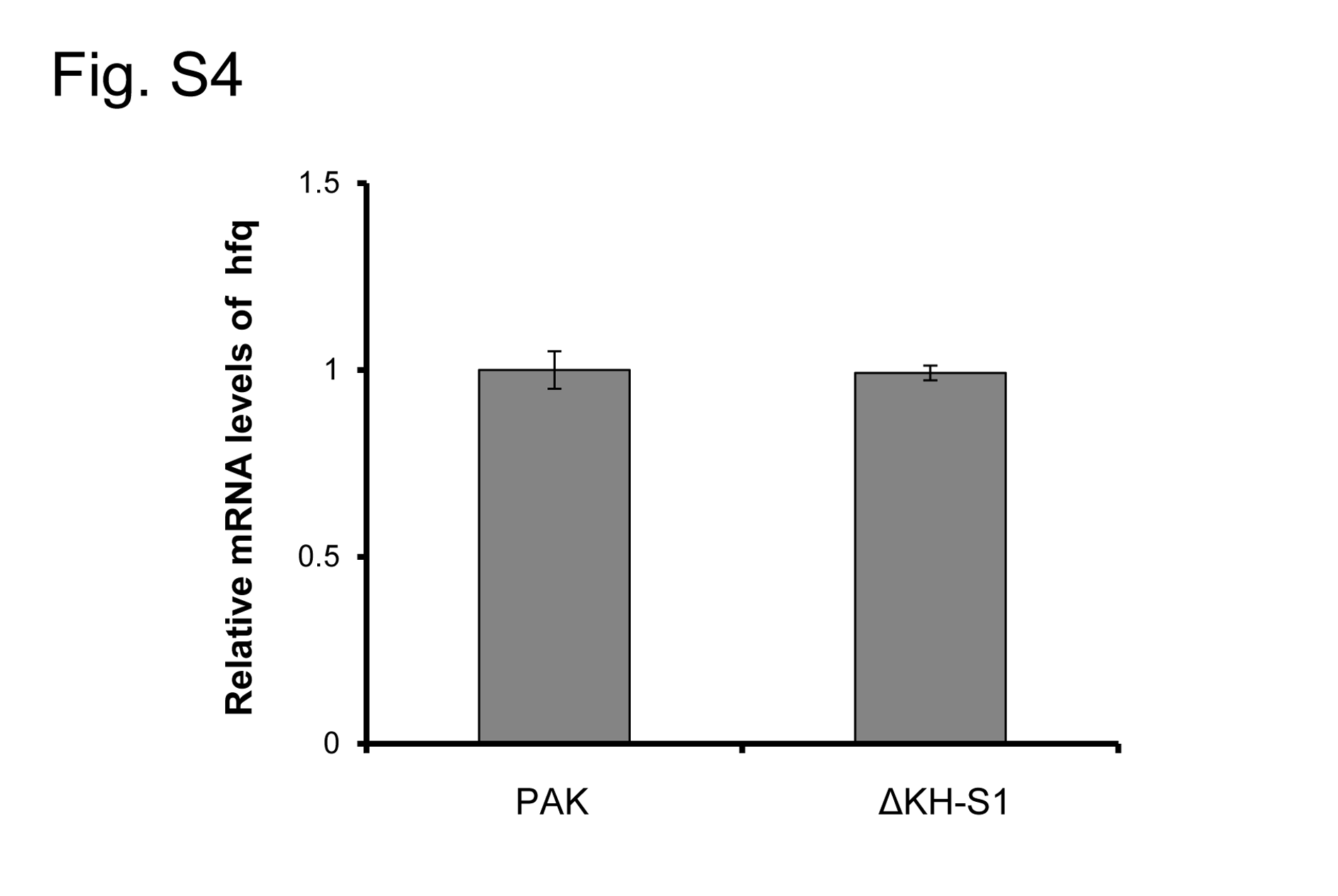

Supplement: Figure S4 — Levels of Hfq mRNA in wild type PAK and the ΔKH-S1 mutant. Bacteria were grown to an OD600 of 1.0. Total RNA from each strain was purified and analyzed with real time PCR. [file Image4.TIF]

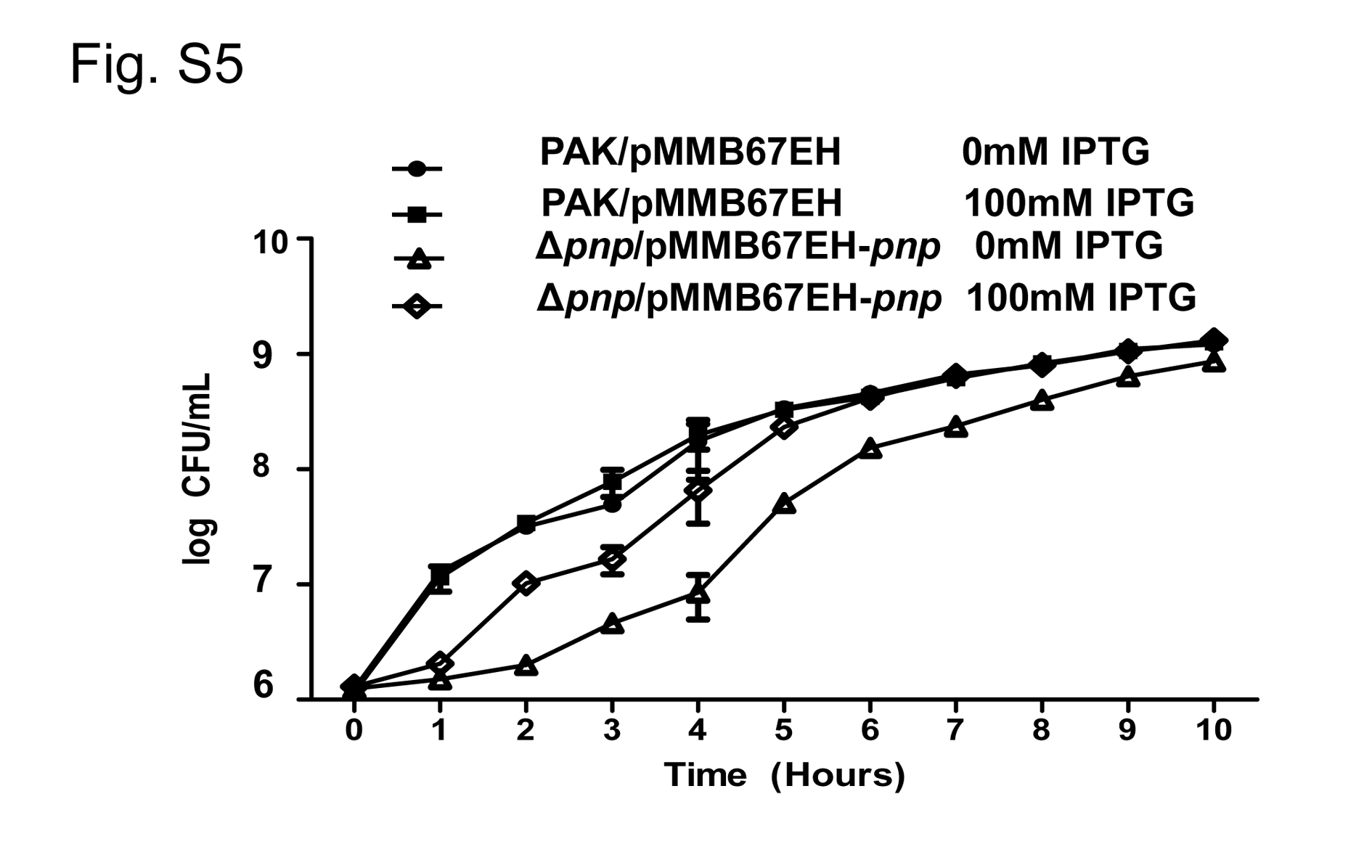

Supplement: Figure S5 — Growth of indicated strains. The wild type PA14 containing pMMB67EH or the Δpnp mutant containing pMMB67EH-pnp was grown in the presence of 100 μM IPTG for 16 h. Each bacterial culture was diluted into fresh LB to an OD600 of 0.03. The bacteria were then grown in the absence of presence of 100 μM IPTG with agitation. The bacteria concentration was determined by serial dilution and plating every hour. [file Image5.TIF]
